# UA-DMSPE Determination of Cu(II), Cd(II), and As(III) in Water, Soil, and Tomato Using a Novel Thiosemicarbazone Sorbent: ICP-OES Performance with DFT Characterization and Antimicrobial-Target Docking

**DOI:** 10.1021/acsomega.5c08559

**Published:** 2025-12-30

**Authors:** Serkan Öncüoğlu

**Affiliations:** Department of Chemistry, 37508 Faculty of Science Dokuz Eylul University, Izmir 35210, Turkey

## Abstract

Heavy metal pollution remains a critical global issue due to the toxic and bioaccumulative nature of elements, such as Cu­(II), Cd­(II), and As­(III). Their occurrence in water, soil, and food products—particularly vegetables—poses serious ecological and health risks. In this work, a novel thiosemicarbazone (TSC) derivative was synthesized and structurally characterized, which has not been previously reported in the literature. The ligand was covalently immobilized onto a silica-based sorbent and applied in an ultrasound-assisted dispersive microsolid phase extraction (UA-DMSPE) protocol for the selective extraction and preconcentration of Cu­(II), Cd­(II), and As­(III). The method was validated on real samples, including irrigation water, agricultural soil, and tomato matrices collected from the Gediz River Basin (Türkiye), a region of intensive agricultural activity. Coupled with ICP-OES, the developed procedure provided high sensitivity, low detection limits, and reliable performance with minimal matrix effects. In addition, density functional theory (DFT) calculations were performed to probe the electronic features and reactivity of the ligand, while molecular docking studies explored its potential antimicrobial interactions. This integrated approach highlights the multifunctionality of the TSC ligand, offering both environmental monitoring capabilities and prospective biomedical applications. Overall, the proposed method demonstrates analytical robustness, environmental compatibility, and a strong potential for routine trace metal surveillance in complex matrices.

## Graphical abstract

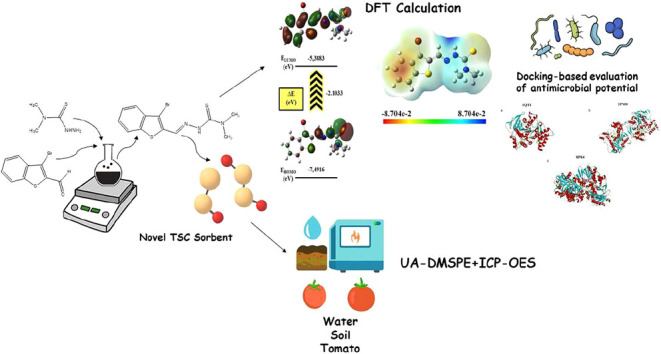

## Introduction

Heavy metals such as Cu­(II), Cd­(II), and As­(III) pose significant threats to both the environment and human health due to their toxicity, bioaccumulation potential, and persistence in ecosystems. Especially cadmium and arsenic, even at trace concentrations, are known to exert nephrotoxic, neurotoxic, and carcinogenic effects through long-term exposure.^1–6^ Therefore, their accurate, sensitive, and selective determination in natural and drinking water sources is one of the critical challenges in environmental monitoring. Although spectroscopic techniques such as ICP-OES and ICP-MS offer high sensitivity, the ultratrace levels of these metals in complex environmental matrices necessitate effective preconcentration strategies to ensure reliable quantification.^7–10^ Among various sample preparation techniques, dispersive microsolid-phase extraction (DMSPE) has gained considerable attention due to its rapid phase interaction, low solvent consumption, and elimination of elution steps.^11–21^

In addition to aquatic matrices, the accumulation of heavy metals such as Cu­(II), Cd­(II), and As­(III) in soil poses serious ecological and agricultural threats.^22–24^ However, the accurate determination of heavy metals in soils is complicated by strong adsorption to heterogeneous mineral phases, binding to humic substances, and site-specific variability, all of which can obscure true analyte concentrations and hinder reproducibility. Vegetables cultivated in metal-contaminated soils such as tomatoes can absorb these elements and introduce them into the human food chain either directly or through animal-derived products. This exposure route may result in severe health effects, including gastrointestinal and respiratory disorders, liver toxicity, and even cancer.^25–27^ Moreover, the trace metal balance in soil, including essential elements such as Zn, Fe, and Cu, significantly affects plant health and crop productivity. While micronutrients are vital for growth, excessive levels of certain metals (e.g., Al, Fe) may negatively impact soil fertility.^28–32^ Hence, trace metal analysis in soil is crucial not only for toxicological assessment but also for sustainable agricultural practices.^33–35^

Plant-based matrices, particularly edible vegetables, are also valuable indicators of environmental contamination. Nevertheless, accurate monitoring in plant tissues remains challenging due to multistep pretreatment requirements (washing, drying, grinding, digestion) and the high organic content of the matrix, which can cause spectral interferences and analyte loss during preparation. Major elements like Ca, K, and Mg, along with trace essential elements such as Fe, Zn, Mn, Cu, and Se, are vital for human metabolism.^36–39^ However, their excess or deficiency may lead to various disorders, and toxic elements such as Cd, Pb, As, and Hg can pose serious health risks even at very low concentrations.^40–44^ Inductively coupled plasma optical emission spectroscopy (ICP-OES) remains a leading technique for elemental analysis in plant samples due to its multielement detection, broad linear range, and low detection limits.^45–49^ Yet, accurate analysis of solid plant matrices like vegetables requires multistep sample preparation including washing, drying, grinding, acid digestion, and filtration before instrumental measurement.^50^ In line with the principles of green analytical chemistry, recent efforts focus on minimizing reagent use, simplifying procedures, and improving environmental sustainability in such analytical workflows.^51–54^

A critical factor in DMSPE is the selection of a suitable chelating agent that offers fast complexation, aqueous solubility, and stability over a wide pH range. In this context, thiosemicarbazones (TSCs) have emerged as highly effective ligands due to their polydentate nature and versatile coordination behavior, especially toward soft metal ions such as Cu­(II), Cd­(II), and As­(III).^55–65^ Literature reveals that TSC ligands not only improve analytical performance in metal extraction but also display significant biological activities including antibacterial, antifungal, antituberculosis, and anticancer properties.^66–68^ Moreover, the incorporation of nitrogen-containing heterocycles, such as pyrazole rings, into TSC structures has been shown to enhance both coordination capacity and pharmacological potential.^69^ These structural features increase cell membrane permeability and biological target interaction, broadening their application in medicinal chemistry.^70–76^ TSC-based metal complexes, particularly with Cu­(II), Ni­(II), Co­(II), Pt­(II), and Ru­(II), have exhibited promising results against various bacterial strains and tumor cell lines.^77–79^

Recent studies have also demonstrated the potential of nanoparticle-functionalized sorbents such as Fe_3_O_4_ magnetic cores coated with poly­(8-hydroxyquinoline) for the selective and efficient extraction of Cu­(II) from complex matrices like soil, tomato, and water samples.^80^ These materials offer excellent surface area, tunable selectivity, and low detection limits, while their magnetic responsiveness facilitates rapid separation without centrifugation.^81–84^ The selection of water, soil, and tomato matrices was made to encompass distinct yet complementary exposure pathways: water as a direct route for both human consumption and agricultural applications, soil as the principal environmental reservoir and source of contamination, and tomato as a representative edible crop widely consumed for its capacity to accumulate trace metals from contaminated soils.

In this study, I report the synthesis and structural characterization of a novel thiosemicarbazone derivative not previously described in the literature. The synthesized ligand was covalently immobilized onto a silica-based sorbent and applied in the UA-DMSPE procedure for the selective extraction and enrichment of Cu­(II), Cd­(II), and As­(III) from environmental water, soil, and tomato samples, followed by quantification via ICP-OES. In this regard, the UA-DMSPE approach developed in this study minimizes sample handling steps, reduces matrix interferences, and enhances recovery efficiency, thereby addressing many of the limitations commonly associated with soil- and plant-based matrices. The novelty of this work lies in combining a newly designed TSC derivative with UA-DMSPE to achieve a high recovery at trace levels with improved reproducibility. Furthermore, the integration of DFT calculations and docking studies provides a unique interdisciplinary perspective, reinforcing both the environmental monitoring applications and the potential biomedical relevance of the ligand.

## Experimental Section

### Chemicals and Reagents

All reagents and solvents employed for the synthesis of the thiosemicarbazone derivative were of analytical grade and were obtained from Merck, Fluka, or Riedel-de Haën. Silica gel (70–230 mesh) was used as the support material. Metal salts utilized for analytical studies, including CuCl_2_, CdCl_2_, and NaAsO_2_, were purchased from Merck, Sigma-Aldrich, or Alfa Aesar. Stock and standard solutions of Cu­(II), Cd­(II), and As­(III) were prepared in ultrapure water and stored at +4 °C. Working solutions were freshly prepared on a daily basis through appropriate dilutions of the stock solutions. Melting points of the synthesized compounds were determined in sealed capillaries by using a digital electrothermal melting point apparatus (Gallenkamp).

The structural characterization of the synthesized compound was carried out using ^1^H NMR spectroscopy on a high-resolution Bruker WH-400 Fourier Transform NMR spectrometer. FTIR spectra were recorded by using a PerkinElmer Spectrum BX-II instrument. Elemental compositions (C, H, N, and S) were determined by using a LECO CHNS-O-9320 elemental analyzer. Surface morphology and elemental composition of the sorbent were examined by scanning electron microscopy (SEM) coupled with energy-dispersive X-ray spectroscopy (EDX), using a Zeiss Evo HD15 system equipped with a tungsten electron source.

Ultrasound-assisted extraction procedures were performed in a Bandelin ultrasonic bath. Ultrapure water utilized throughout the experiments was supplied by a Thermo Scientific Smart2Pure Pro system. Quantitative analysis of metal ions was conducted by using a Varian 710-ES inductively coupled plasma optical emission spectrometer (ICP-OES). The emission wavelengths employed for the determination of Cu­(II), Cd­(II), and As­(III) were 324.75, 228.80, and 193.70 nm, respectively.

### Sample Preparation

Accurate determination of trace metals in complex matrices, such as environmental water, soil, and plant tissues, requires carefully designed sample preparation steps to ensure reliability, reproducibility, and minimal matrix interferences.

#### Water Samples

Surface water and irrigation water samples were collected from various agricultural zones within the Gediz River Basin (Türkiye) using precleaned polyethylene bottles. The samples were filtered through 0.45 μm membrane filters to remove suspended particulates and stored at 4 °C until analysis. Prior to extraction, the pH of each water sample was adjusted to 6.0 using dilute HCl or NaOH solutions to optimize metal–ligand complexation during the UA-DMSPE procedure.

#### Soil Samples

Soil specimens were obtained from the top 0–15 cm layer of agricultural fields within the same region. After the sample was air-dried under controlled laboratory conditions, visible debris and plant residues were removed. The dried soils were ground using a mortar and passed through a 0.5 mm sieve to obtain a uniform particle size. For digestion, 1.0 g of each soil sample was treated with 6 mL of concentrated HNO_3_ and 2 mL of 30% H_2_O_2_ in a Teflon digestion vessel. The mixture was subjected to a stepwise heating program by using a microwave-assisted system until complete mineralization was achieved. The digests were cooled, filtered, and diluted to 30 mL with ultrapure water before metal analysis.

#### Tomato Samples

Tomato fruits were collected from selected agricultural sites within the Gediz Basin, with only the edible portions retained for trace metal determination. To eliminate adhering soil particles and atmospheric contaminants, samples were first rinsed thoroughly with tap water, followed by multiple washes with deionized water.^85,86^ The cleaned material was then oven-dried at 60 °C for 24–48 h until a constant weight was achieved, ensuring complete moisture removal without inducing thermal degradation of target analytes.^87–90^ The dried tissues were subsequently ground to a homogeneous fine powder using an agate mortar and pestle, and the powder was stored in airtight polyethylene containers to prevent contamination and moisture uptake. For sample digestion, 0.5 g of the powdered material was subjected to wet acid decomposition with 6 mL of concentrated HNO_3_ and 2 mL of 30% H_2_O_2_ in a microwave-assisted digestion system, employing a five-step temperature ramping program.^91,92^ After being cooled to room temperature, the digests were filtered and quantitatively diluted to 25 mL with ultrapure water prior to ICP-OES analysis.

The overall preparation strategy was optimized according to the principles of green analytical chemistry, with the objective of minimizing reagent consumption, maximizing analyte recovery, and improving compatibility with complex biological matrices.^93–95^

### Synthesis of (*E*)-2-((3-Bromobenzo­[*b*]­thiophen-2-yl)­methylene)-*N*,*N*-dimethylhydrazine-1-carbothioamide

(*E*)-2-((3-bromobenzo­[*b*]­thiophen-2-yl)­methylene)-*N*,*N*-dimethylhydrazine-1-carbothioamide was synthesized following a reported procedure.^96^ It was obtained via a condensation reaction between *N*,*N*-dimethylhydrazinecarbothioamide (0.06 g, 0.5 mmol) and 3-bromobenzo­[*b*]­thiophene-2-carbaldehyde (0.121 g, 0.5 mmol) in 20 mL of absolute ethanol. To facilitate the reaction, 2–3 drops of glacial acetic acid were added, and the mixture was refluxed under constant stirring for 5 h. The progress was monitored by thin-layer chromatography (TLC). Upon completion, the mixture was cooled to room temperature, which afforded a yellow crystalline solid. The product was filtered, washed successively with cold methanol and diethyl ether, and recrystallized from dichloromethane to afford the purified ligand. Yield: 76%; yellow powder; mp 203 °C (Figure 1).

**Figure 1:**
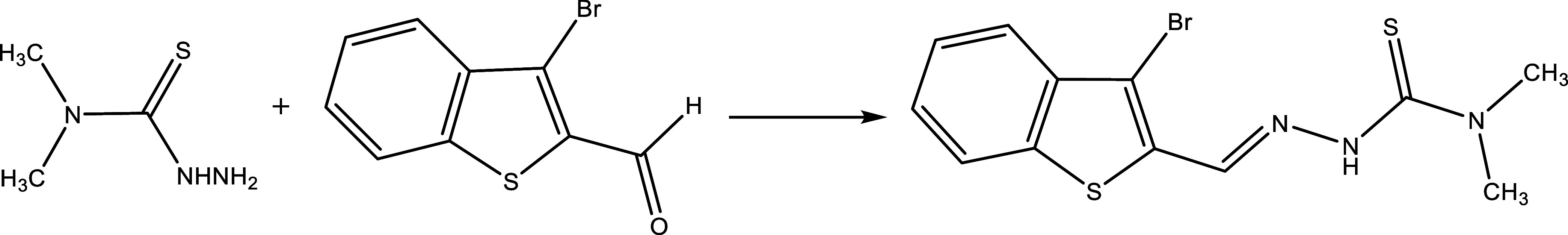
Synthesis of (*E*)-2-((3-bromobenzo­[*b*]­thiophen-2-yl)­methylene)-*N*,*N*-dimethylhydrazine-1-carbothioamide.

### Preparation of Sorbent

Prior to functionalization, the silica gel was pretreated with 0.5 M HNO_3_ to remove residual contaminants. After thorough acid washing, it was rinsed repeatedly with deionized water until neutral pH was reached. Subsequently, 1.5 g of activated silica was mixed with 15 mL of a 1 × 10^–4^ mol L^–1^ solution of the synthesized thiosemicarbazone (TSC) derivative (*E*)-2-((3-bromobenzo­[*b*]­thiophen-2-yl)­methylene)-*N*,*N*-dimethylhydrazine-1-carbothioamide in chloroform. The mixture was stirred at ambient temperature for 24 h to facilitate immobilization. The resulting material was collected by vacuum filtration using a sintered glass funnel. To remove any physically adsorbed ligand, the functionalized silica was washed thoroughly with chloroform and subsequently with ultrapure water. The final product was dried under vacuum and stored in a desiccator until further use.^97^

### Ultrasound-Assisted Dispersive Microsolid-Phase Extraction Procedure (UA-DMSPE)

An aliquot of 10 mL of either a 250 μg L^–1^ standard metal mixture or a digested soil sample (prepared by microwave-assisted acid digestion) was transferred into a polypropylene centrifuge tube containing 40 mg of TSC-functionalized silica sorbent. The suspension was sonicated at ambient temperature for 15 min to ensure efficient interaction between the analytes and the sorbent surface. It was then centrifuged at 5000 rpm for 10 min, and the supernatant was decanted. For desorption of the retained metal ions, 0.5 mL of 1 mol L^–1^ HNO_3_ was added to the sediment, followed by sonication for 10 min. The mixture was centrifuged again at 5000 rpm for 10 min. The resulting supernatant was passed through a 0.45 μm membrane filter to remove residual particulates prior to analysis. The filtrate was analyzed by ICP-OES for the quantification of Cu­(II), Cd­(II), and As­(III) ions.^98^

### Protein Modeling

Three-dimensional structures of the proteins (PDB IDs: 3KFD, 5YEI, and 6Y2E) were constructed using the BIOVIA Discovery Studio. During the modeling process, templates providing the highest sequence coverage and the best resolution were selected to ensure structural accuracy. As shown in Figure 2, the resulting protein models exhibited distinct conformations, which can be attributed to differences in amino acid composition and intramolecular interactions.

**Figure 2:**
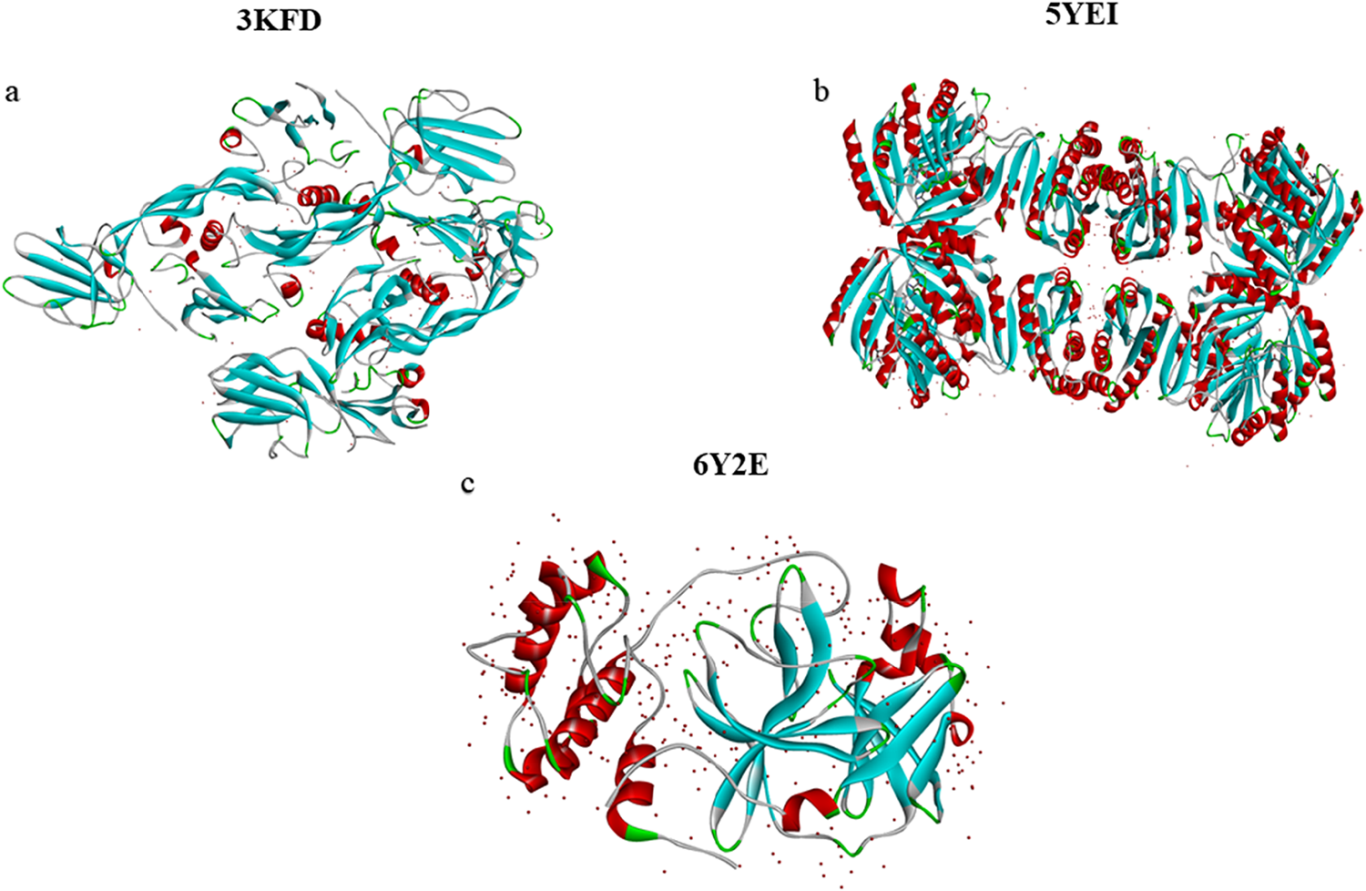
Modeled 3D structures of (a) 3KFD, (b) 5YEI, and (c) 6Y2E using BIOVIA discovery studio visualizer 2024.

### Molecular Docking

Following the structural modeling of 3KFD, 5YEI, and 6Y2E proteins, molecular docking analyses were carried out using CB-Dock2,^99^ with a synthesized thiosemicarbazone derivative employed as the ligand. CB-Dock2 utilizes a cavity-centered prediction algorithm to identify potential binding regions and estimate binding affinities. For each protein, five binding pockets were detected and subsequently ranked based on their calculated binding energies, allowing prioritization of the most favorable interaction sites.

### Density Functional Theory (DFT)

Density functional theory (DFT) calculations were performed to investigate the electronic properties of the synthesized thiosemicarbazone ligand.^100^ Molecular sketching and structural modeling were conducted using ChemDraw and Avogadro, while geometry optimizations and electronic structure calculations were carried out with GaussView 5.0 and Gaussian 09W.^101–103^ The optimized geometries were obtained at the B3LYP/6–311G­(d,p) level of theory for C, N, O, and H atoms to ensure stable configurations and reliable electronic descriptions.

Molecular electrostatic potential (MEP) maps were generated to visualize the electrophilic and nucleophilic regions of the molecule. In these maps, electron-deficient regions favorable for nucleophilic attack are shown in blue, whereas electron-rich regions prone to electrophilic attack are represented in red; neutral zones appear in green.^104^

Frontier molecular orbital (FMO) analysis was also performed, focusing on the highest occupied molecular orbital (HOMO) and the lowest unoccupied molecular orbital (LUMO).^105^ The HOMO indicates the electron-donating ability, while the LUMO reflects the electron-accepting tendency of the molecule.^106,107^ The HOMO–LUMO energy gap (Δ*E*), defined as the difference between these orbitals, serves as a key descriptor of chemical reactivity and stability. A larger Δ*E* corresponds to higher stability and lower reactivity, whereas a smaller Δ*E* suggests enhanced reactivity.

Based on FMO results, several global reactivity descriptors—including ionization potential (*I*), electron affinity (EA), electronegativity (χ), chemical hardness (η), softness (σ), chemical potential (μ), and electrophilicity index (ω)—were calculated using standard equations (eqs 1–8) reported in the literature^107,108^

1 $${\Delta E}_{\mathrm{gap}}=E_{\mathrm{LUMO}}-E_{\mathrm{HOMO}}$$

2 $$I=-E_{\mathrm{HOMO}}$$

3 $$\mathrm{EA}=-E_{\mathrm{LUMO}}$$

4 $$\chi =\frac{I+\mathrm{EA}}{2}$$

5 $$\eta =\frac{E_{\mathrm{LUMO}}-E_{\mathrm{HOMO}}}{2}$$

6 $$\sigma =\frac{1}{2\eta }$$

7 $$\mu =\frac{E_{\mathrm{HOMO}}+E_{\mathrm{LUMO}}}{2}$$

8 $$\omega =\frac{\mu^{2}}{2\eta }$$

## Results and Discussion

### Characterization of Si-Carb-Formazan

Structural characterization of the synthesized silica-based sorbent modified with a thiosemicarbazone (TSC) ligand was conducted by using FTIR spectroscopy and SEM-EDX analyses. As illustrated in Figure 3, the FTIR spectra provided clear evidence for the presence of both the silica support and the organic ligand. In the spectrum of unmodified silica (red), a broad absorption around 3436 cm^–1^ was attributed to O–H stretching vibrations of surface silanol groups, while a distinct peak near 1090 cm^–1^ indicated the asymmetric stretching of Si–O–Si bonds. Additional bands between 966 and 808 cm^–1^ were assigned to bending vibrations associated with Si–O and Si–OH functionalities.

**Figure 3:**
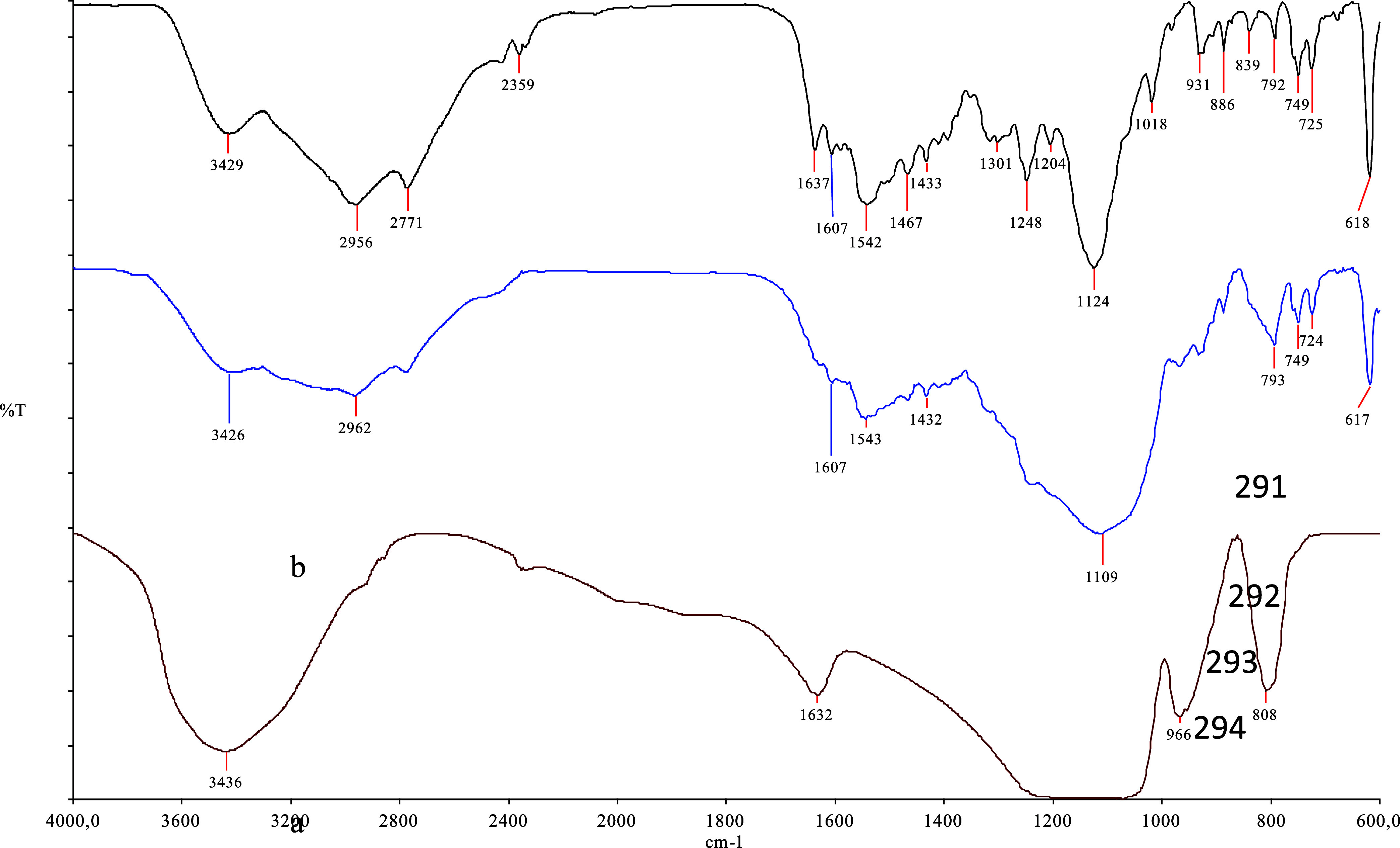
Comparative FTIR spectra of the TSC ligand (black), functionalized silica material (blue), and unmodified silica (red) are shown from top to bottom, respectively.

The pure TSC ligand spectrum (black) revealed a broad band at 3429 cm^–1^, corresponding to N–H stretching vibrations. A strong absorption at 1542 cm^–1^ confirmed the presence of the imine group (C=N), a key structural feature of the TSC. Moreover, the peaks at 2956 and 2771 cm^–1^ were consistent with aliphatic C–H stretching modes, and the range between 1248 and 1018 cm^–1^ showed characteristic C–N stretching vibrations.

In the spectrum of the functionalized silica material (blue), absorption features from both the silica matrix and the organic ligand were observed, indicating successful covalent grafting. The persistent presence of the imine C=N band at 1543 cm^–1^ demonstrated that the TSC retained its structural identity postimmobilization. The broad signal at 3426 cm^–1^ suggested overlapping of the O–H and N–H stretching vibrations from the silica and the ligand moieties. Furthermore, the intense band at 1109 cm^–1^ confirmed the structural integrity of the silica framework through Si–O–Si vibrations, and the peaks at 2962 and 2770 cm^–1^ supported the presence of aliphatic C–H groups associated with the ligand. Altogether, these spectroscopic observations conclusively verified the successful functionalization of the silica surface with the TSC ligand via chemical bonding interactions.

Morphological and elemental characteristics of the TSC-functionalized silica sorbent were evaluated both before and after metal ion adsorption using scanning electron microscopy (SEM), energy-dispersive X-ray spectroscopy (EDX), and elemental mapping techniques. As depicted in Figure 4, the SEM micrograph obtained prior to metal binding revealed a relatively uniform and porous surface topology. Following the adsorption process, noticeable morphological changes were observed, including increased surface roughness and particle agglomeration, indicating the interaction between the sorbent and metal ions.

**Figure 4:**
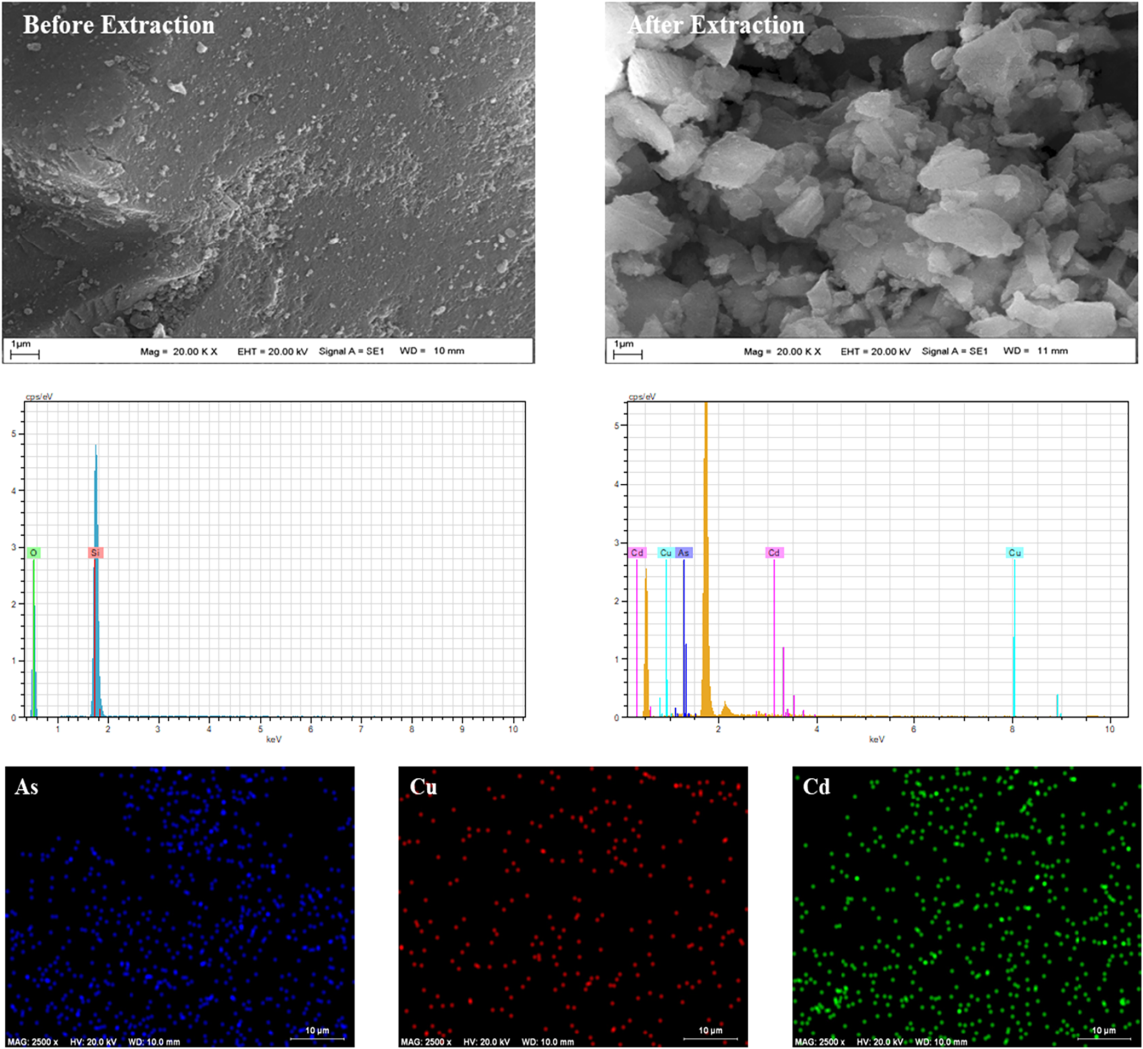
SEM images and EDX spectra of the sorbent before and after metal sorption.

Furthermore, EDX spectral analysis of the pristine sorbent confirmed the presence of only silicon (Si) and oxygen (O), consistent with the silica matrix. However, after exposure to metal-containing samples, new peaks corresponding to arsenic (As), cadmium (Cd), and copper (Cu) appeared in the EDX spectrum, demonstrating that these metal ions were successfully captured by the sorbent. Complementary EDX mapping further confirmed that these metal ions were uniformly distributed across the surface of the material, providing visual evidence of effective and homogeneous adsorption. Collectively, these findings validate the selectivity and efficiency of the prepared sorbent for the adsorption of As­(III), Cd­(II), and Cu­(II) ions from complex matrices.

C_12_H_12_BrN_3_S_2_: Calc. %C: 42.11; H, 3.53; N, 12.28; S, 18.73. Found: %C: 41.81; H, 3.42; N, 12.17; S, 18.75. FTIR (KBr pellet) ν/cm^–1^ 3429 (N–H), 1542 (C=N), 1124 (N–N), 839 (C=S), 792 and 725 (thiophen and phenyl ring stretching). ^1^H NMR (400 MHz, DMSO-d6) data [δ 11.14 (s, 1H), 8.33 (s, 1H), 7.97–7.89 (m, 2H, ArH), 7.50–7.39 (m, 2H, ArH), 3.23 (s, 6H, N­(CH_3_)_2_)] are correctly cited as Figure 5, whereas the ^13^C NMR (100 MHz, chloroform-d) data [δ: 173.89, 143.82, 137.17, 134.85, 131.50, 127.95, 125.69, 125.57, 122.63, 116.97, 40.18 (2 × C)] are cited as Figure 6.

**Figure 5:**
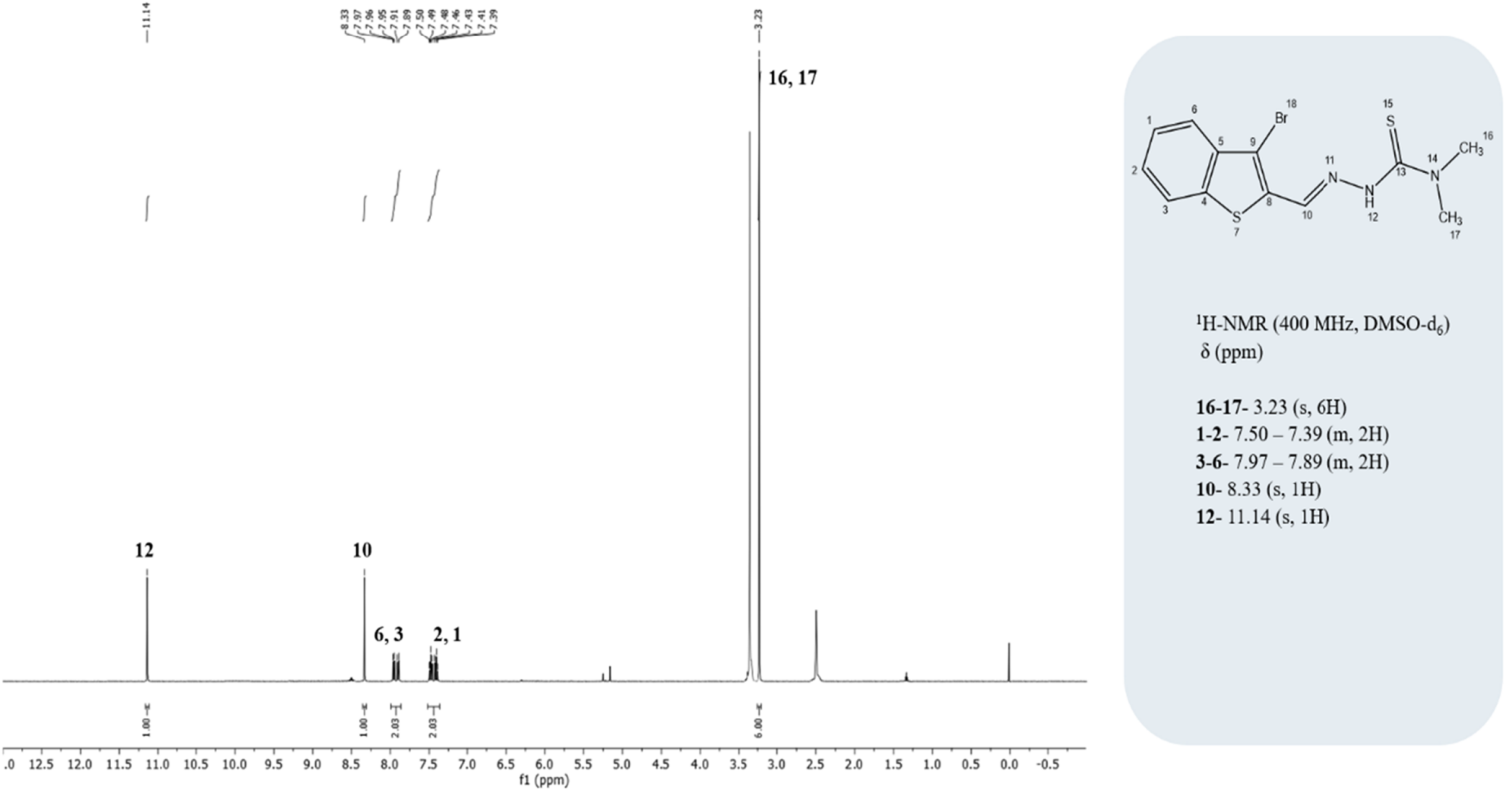
^1^H NMR spectrum of the synthesized thiosemicarbazone (TSC) ligand.

**Figure 6:**
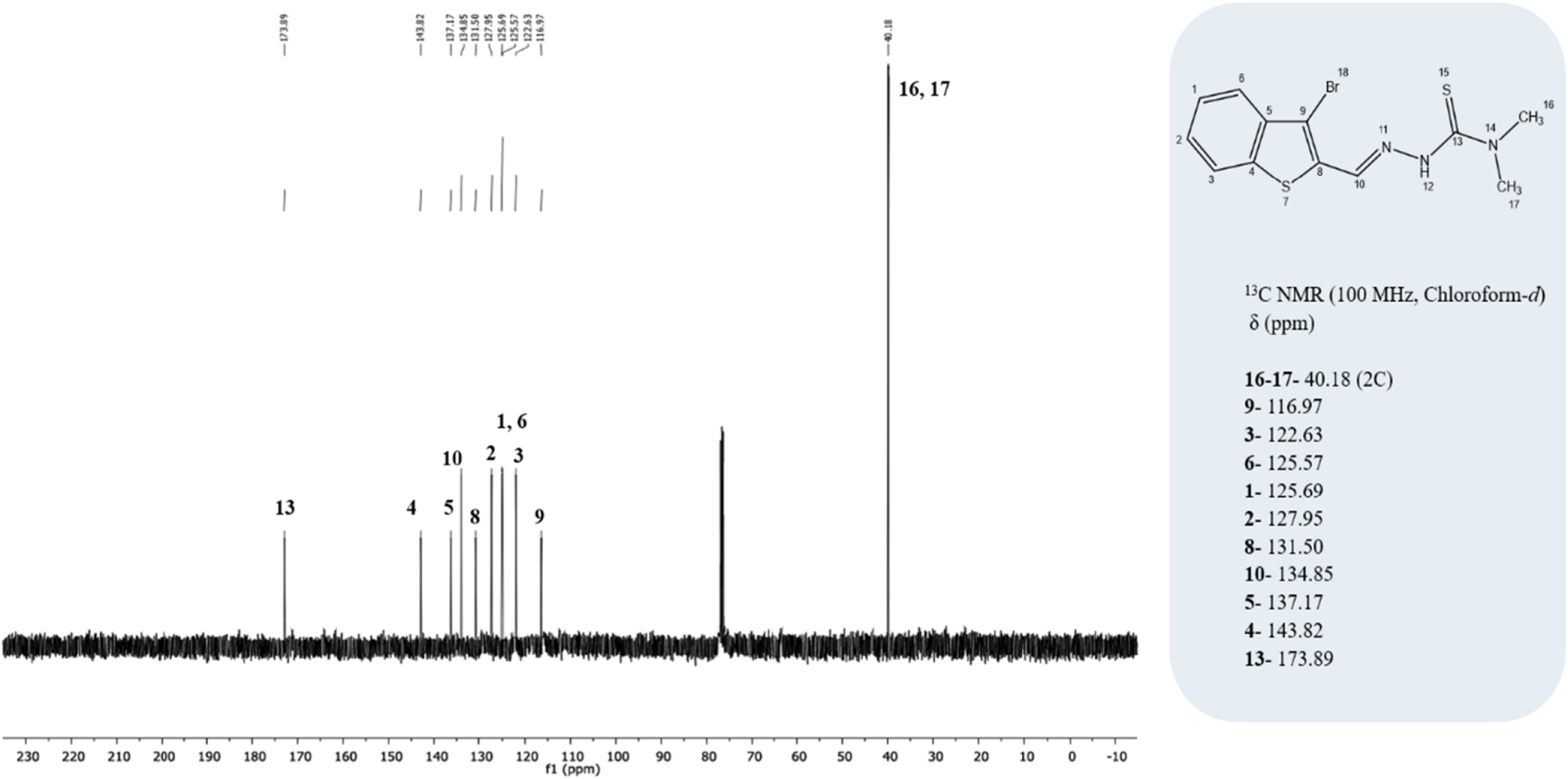
^13^C NMR spectrum of the synthesized thiosemicarbazone (TSC) ligand.

### Method Optimization Studies

For improved efficiency of the ultrasound-assisted dispersive microsolid phase extraction (UA-DMSPE) procedure, experimental parameters were systematically optimized. The optimization considered key variables, including sample pH, sorbent dosage, extraction time, and the type and volume of the eluent. A one-factor-at-a-time (OFAT) approach was applied, where each variable was altered individually while others were held constant, enabling the independent evaluation of its effect on Cu­(II), Cd­(II), and As­(III) recovery. The conditions yielding the highest recovery efficiencies were selected as optimal. This classical OFAT strategy provided a straightforward and effective framework for optimizing the UA-DMSPE method without the application of complex statistical or multivariate approaches.

### Effect of Ligand Concentration

In the UA-DMSPE technique, the efficiency of metal ion extraction is highly influenced by the amount of ligand immobilized on the sorbent surface. As supported by previous reports, insufficient ligand concentration may lead to poor complexation with target metal ions, whereas excessive ligand density may result in steric hindrance or oversaturation effects, ultimately reducing sorption performance. To investigate this, sorbents were prepared by functionalizing 1 g of activated silica with 10 mL Schiff base ligand solutions at varying concentrations (1 × 10^–5^, 5 × 10^–5^, 1 × 10^–4^, and 5 × 10^–4^ mol L^–1^). The resulting materials were applied in UA-DMSPE procedures, and the retention efficiencies of Cu­(II), Cd­(II), and As­(III) were evaluated.

As illustrated in Figure 7, increasing the ligand concentration led to a notable improvement in metal recoveries, reaching maximum values of 91.3% for Cu­(II), 91.0% for Cd­(II), and 89.5% for As­(III) at 1 × 10^–4^ mol L^–1^. Beyond this concentration, a slight decline in recovery was observed, likely due to excessive ligand coverage, causing diffusion or steric limitations. Therefore, the sorbent prepared using a ligand concentration of 1 × 10^–4^ mol L^–1^ was deemed optimal and selected for subsequent extractions.^109^

### Eluent Selection and Volume Optimization

To investigate the influence of eluent type on the desorption efficiency, several acid solutions were evaluated, including nitric acid (HNO_3_) and hydrochloric acid (HCl) at concentrations of 0.5 and 1 M, prepared in both water and methanol. As illustrated in Figure 7, the highest metal recovery (59.1%) was obtained using 1 M HNO_3_ in water, indicating its superior elution capacity compared to those of other eluents. This enhanced efficiency can be attributed to the stronger oxidative nature of nitric acid and its more effective disruption of metal–ligand interactions on the sorbent surface. In contrast, the use of HNO_3_ in methanol or lower concentrations of HCl resulted in significantly reduced recoveries, likely due to weaker acid strength or less effective solvent penetration. These findings support the selection of 1 M aqueous HNO_3_ as the most suitable eluent for the UA-D-mSPE procedure, in line with similar observations in prior studies.^110,111^

The volume of the eluent plays a crucial role in ensuring complete desorption of analytes from the sorbent without causing analyte dilution. In this study, varying volumes (0.25, 0.5, 1.0, 1.5, and 2.0 mL) of 1 M HNO_3_ in water were tested, and their influence on the recovery efficiency of Cu­(II), Cd­(II), and As­(III) was recorded. As depicted in Figure 7, the recovery values increased steadily with eluent volume up to 0.5 mL, after which a slight decline was observed. This trend is likely due to improved eluent–sorbent interaction at optimal volume, promoting efficient desorption, whereas excessive eluent volumes may dilute the analytes or exceed the sorbent’s release capacity. Based on these results, 0.5 mL of 1 M HNO_3_ was selected as the optimal eluent volume for further experiments, balancing maximum recovery and minimal dilution effect.

**Figure 7:**
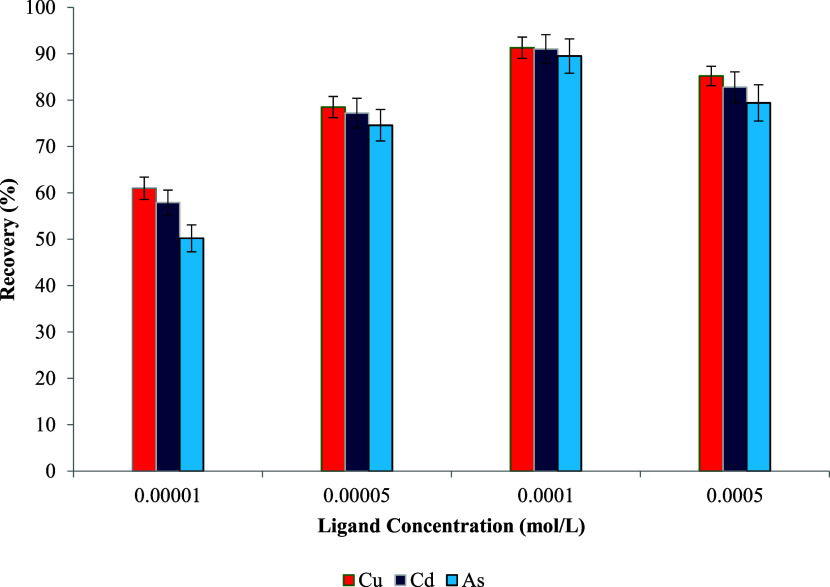
Effect of ligand concentration (*n* = 3).

### pH Optimization

The influence of sample pH on the recovery of Cu­(II), Cd­(II), and As­(III) ions was thoroughly investigated over a pH range of 3–10 to determine the optimal extraction conditions. As shown in Figure 8, the recovery efficiencies of all three analytes were significantly reduced at low pH values (3–4), which is likely due to the high concentration of H^+^ ions competing with metal ions for active binding sites on the sorbent surface. At pH 3, the recovery rates were 37.0% for Cu­(II), 34.0% for Cd­(II), and 31.0% for As­(III).

**Figure 8:**
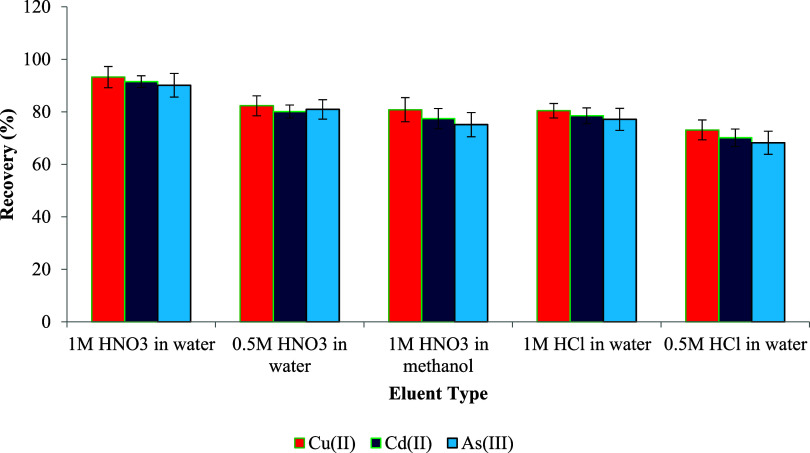
Effect of eluent type on metal ion recovery (*n* = 3).

A substantial increase in extraction efficiency was observed between pH 5 and 7, with the highest recoveries obtained at pH 7: Cu­(II) 94.0%, Cd­(II) 92.1%, and As­(III) −89.6%. This improvement is attributed to the optimal deprotonation of the ligand functional groups, which enhances their chelation capacity. Notably, this pH also aligns with the natural pH of the sample solutions, thus, eliminating the need for pH adjustment prior to extraction.

At higher pH values (8 and 10), a decline in recovery rates was observed. This reduction may be attributed to the formation of insoluble hydroxide species (e.g., Cu­(OH)_2_, Cd­(OH)_2_, As­(OH)_3_), which diminish the free metal ion concentration available for interaction with the sorbent. Therefore, pH 7 was selected as the optimal condition for subsequent UA-DMSPE procedures involving trace metal analysis.^112,113^

### Sorbent Amount Optimization

The quantity of the sorbent plays a pivotal role in determining the extraction efficiency of trace metals in the UA-DMSPE method. As presented in Figure 9, an increase in the sorbent amount from 20 to 50 mg resulted in a steady enhancement in the recovery of Cu­(II), Cd­(II), and As­(III) ions, reaching peak values of 95.0, 94.2, and 92.5%, respectively, at 50 mg. This improvement is attributed to the increased availability of active binding sites, which facilitates more effective complexation between the metal ions and the ligand-functionalized silica surface. However, a slight decline in recovery was observed at 60 mg, possibly due to sorbent particle agglomeration, reduced dispersion, or steric hindrance, which may hinder mass transfer or elution efficiency. These findings underscore the importance of optimizing the sorbent dosage, with 50 mg identified as the ideal amount to maximize analyte retention while avoiding adverse effects linked to excessive sorbent use (Figures 10 and 11).^114^

**Figure 9:**
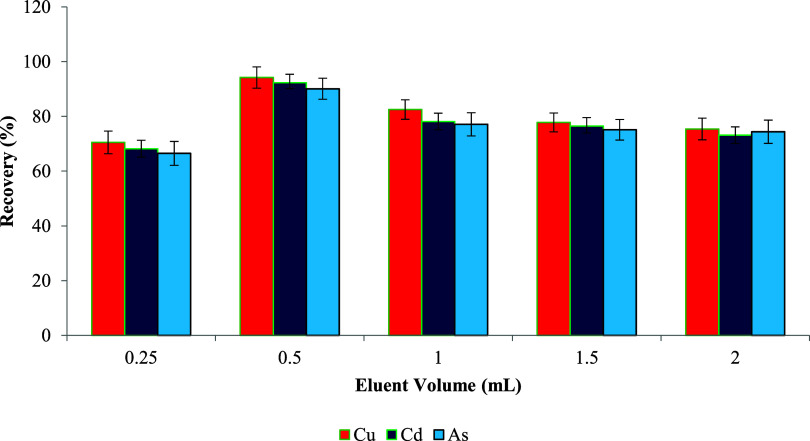
Effect of eluent volume on metal ion recovery (*n* = 3).

**Figure 10:**
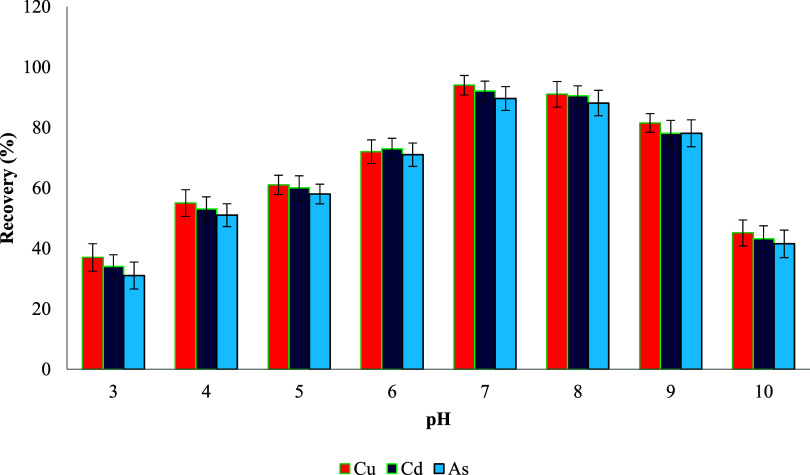
Effect of pH on metal ion recovery (*n* = 3).

**Figure 11:**
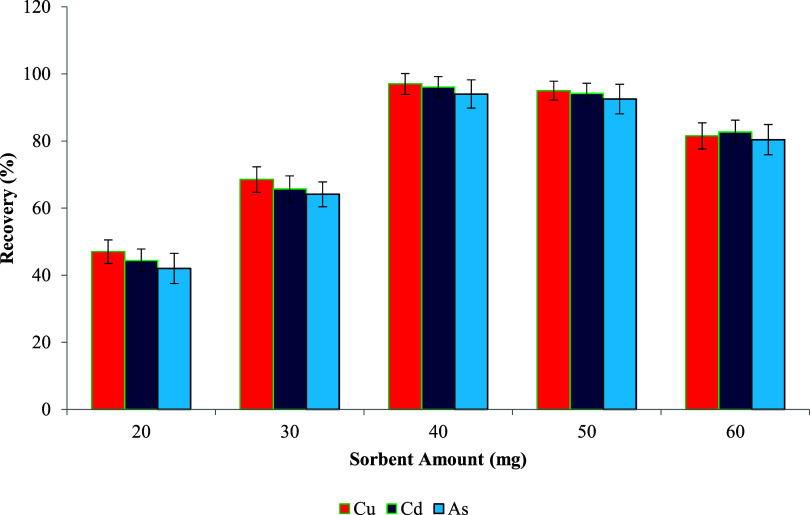
Effect of the sorbent amount on metal ion recovery.

### Extraction Time

Extraction time is a critical factor influencing the efficiency of ultrasound-assisted dispersive microsolid-phase extraction (UA-DMSPE), as it dictates the contact time between analytes and the sorbent surface. As shown in Figure 12, extending the extraction time from 5 to 15 min significantly improved recoveries of Cu­(II), Cd­(II), and As­(III), with maximum values of 99.0, 96.0, and 95.2%, respectively, observed at 15 min. This enhancement can be attributed to sufficient contact time for the establishment of a complexation equilibrium between the ligand-functionalized sorbent and the target metal ions.

**Figure 12:**
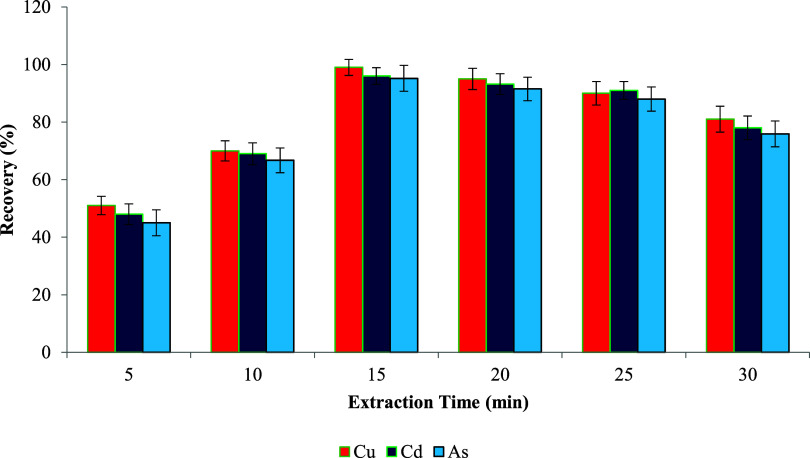
Effect of the extraction time on metal ion recovery.

However, when the extraction time was prolonged to 20 and 25 min, recoveries slightly decreased; for example, Cu­(II) recovery declined to 95.0 and 90.0%, respectively. This reduction may be due to partial desorption of bound metal ions or disruption of metal–sorbent interactions caused by extended ultrasonication, potentially inducing mechanical stress or overheating of the suspension. Therefore, 15 min was identified as the optimal extraction time, offering both high recovery and practical applicability for subsequent analyses.^115^

The optimized experimental parameters for the ultrasound-assisted dispersive microsolid phase extraction (UA-DMSPE) method are summarized in Table 1. The parameters included the ligand concentration for silica functionalization, sample pH, sorbent dosage, extraction time, and eluent type and volume. Each parameter was optimized individually using a one-factor-at-a-time (OFAT) approach to assess its effect on the recovery of Cu­(II), Cd­(II), and As­(III) ions. The final conditions corresponded to those yielding maximum recoveries for all three analytes.

**Table 1:** Optimized Parameters for Metal Ion Extraction

parameter	optimized value
pH	7
sorbent amount (mg)	50
ligand concentration (mol L^–1^)	1 × 10^–4^
extraction time (min)	15
eluent type	1 mol L^–1^ HNO_3_ (in water)
eluent volume (mL)	0.5

### Interference Studies

To evaluate the selectivity of the developed UA-DMSPE method for simultaneous Cu­(II), Cd­(II), and As­(III) extraction, a systematic interference study was performed with various coexisting ions commonly present in environmental matrices such as soil, water, and tomato samples. The study involved spiking standard solutions (250 μg L^–1^ of each target analyte) with potentially interfering cations and anions, including Na^+^, K^+^, Mg^2+^, Ca^2+^, Fe^3+^, Al^3+^, Mn^2+^, Cr^3+^, Zn^2+^, Co^2+^, Ni^2+^, Pb^2+^, Cl^–^, NO_3_^–^, SO_4_^2–^, and PO_4_^3–^. The tolerance limit was defined as the maximum concentration of the interfering species that caused less than 5% deviation in recovery.

As summarized in Table 2, most foreign ions caused negligible interference even at high concentrations (e.g., up to 1000 μg mL^–1^ for Na^+^ and Cl^–^), highlighting the strong selectivity of the TSC-functionalized silica sorbent. For example, Na^+^ and K^+^ at 1000 μg L^–1^ yielded recoveries above 92% for all three analytes. Transition metals such as Fe^3+^, Zn^2+^, and Cr^3+^—typically prone to strong complexation—also exhibited minimal interference, with recoveries consistently above 93% at concentrations of 50–100 μg L^–1^. Notably, in the presence of Zn^2+^, recoveries of 94.8%, 96.5%, and 96.2% were achieved for Cu­(II), Cd­(II), and As­(III), respectively. These findings confirm the excellent resistance of the method to matrix interferences, demonstrating its suitability for trace metal analysis in complex environmental samples.

**Table 2:** Effect of Foreign Ions on the Recovery of Cu­(II), Cd­(II), and As­(III) under Optimized Conditions (*n* = 3)

interfering ion	source salt	tol. limit (μg mL^–1^)	Cu(II) Rec. (%)	Cd(II) Rec. (%)	As(III) Rec. (%)
Na^+^	NaCl	1000	94.2 ± 3.5	95.9 ± 3.0	93.1 ± 2.0
K^+^	KCl	1000	93.0 ± 3.6	95.2 ± 3.2	92.5 ± 3.9
Mg^2+^	Mg(NO_3_)_2_	500	95.8 ± 2.1	93.2 ± 1.9	94.0 ± 2.6
Ca^2+^	CaCl_2_	500	94.1 ± 2.3	94.9 ± 1.8	93.6 ± 2.5
Fe^3+^	FeCl_3_	50	94.6 ± 3.2	93.4 ± 2.8	95.2 ± 1.6
Al^3+^	Al(NO_3_)_3_	50	94.9 ± 1.9	92.6 ± 3.9	96.5 ± 3.1
Mn^2+^	MnCl_2_	100	93.8 ± 1.7	95.4 ± 2.4	92.7 ± 2.8
Cr^3+^	Cr(NO_3_)_3_	100	92.7 ± 3.5	93.6 ± 2.9	93.9 ± 2.5
Zn^2+^	Zn(NO_3_)_2_	50	94.8 ± 1.9	96.5 ± 3.1	96.2 ± 3.5
Co^2+^	Co(NO_3_)_2_	50	94.9 ± 3.7	92.7 ± 1.9	92.6 ± 2.3
Ni^2+^	NiCl_2_	50	94.1 ± 2.1	95.9 ± 2.3	93.5 ± 2.7
Pb^2+^	Pb(NO_3_)_2_	50	92.9 ± 3.4	92.7 ± 4.0	95.7 ± 1.7
Cl^–^	NaCl	1000	92.5 ± 3.4	95.8 ± 3.2	95.9 ± 1.5
NO_3_^–^	NaNO_3_	1000	94.1 ± 1.8	96.3 ± 2.9	93.9 ± 1.6
SO_4_^2–^	Na_2_SO_4_	1000	93.7 ± 2.4	95.8 ± 3.0	96.2 ± 2.4
PO_4_^3–^	Na_3_PO_4_	1000	92.9 ± 3.1	95.9 ± 2.7	95.7 ± 2.6

### Effect of Sample Volume on the Recovery of Cu­(II), Cd­(II), and As­(III) Ions

The sample volume is a key variable in microextraction-based preconcentration techniques as it directly affects both the recovery efficiency and the achievable preconcentration factor. In this study, the effect of sample volume was systematically assessed within the range of 5–25 mL using 250 μg L^–1^ standard solutions. As illustrated in Figure 13, the recoveries for Cu­(II), Cd­(II), and As­(III) initially increased with increasing sample volume, reaching their respective maxima at 20 mL (Cu: 99.7%, Cd: 97.4%, As: 95.7%). This enhancement is likely due to improved mass transfer and prolonged contact between the analytes and the sorbent surface. However, a significant drop in recovery was observed at 25 mL, where recoveries declined to 80.2% (Cu), 79.0% (Cd), and 73.6% (As). This decrease can be attributed to the dilution of analyte concentration and the possible exceedance of the sorbent’s binding capacity, which together reduce the efficiency of analyte–sorbent interactions. Based on these findings, 20 mL was selected as the optimal sample volume, balancing maximum recovery with a suitable preconcentration factor.^116^

**Figure 13:**
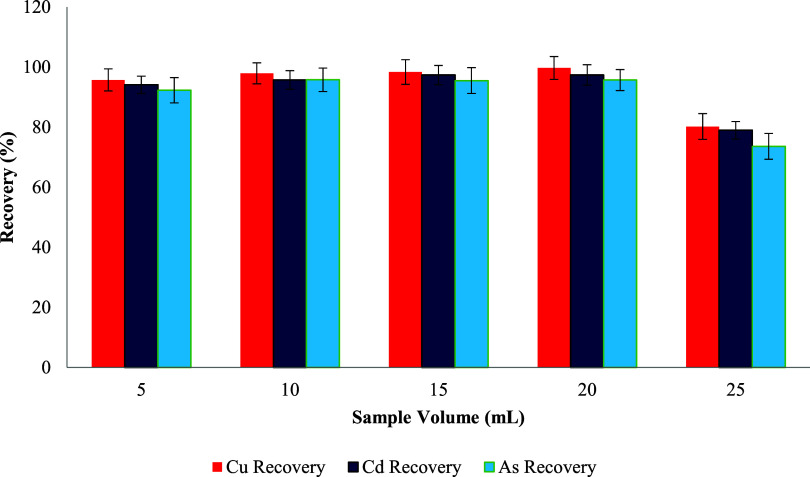
Effect of the sample volume on metal ion recovery.

### Evaluation of Sorbent Stability and Reusability in Successive UA-DMSPE Cycles

The reusability of the thiosemicarbazone-functionalized silica sorbent was systematically investigated to evaluate its operational stability and practical feasibility for repeated extraction cycles. Following each extraction, the sorbent was regenerated by treatment with 1 mL of 1 mol L^–1^ HNO_3_ in an ultrasonic bath for 10 min to ensure complete desorption of Cu­(II), Cd­(II), and As­(III) ions. The regenerated sorbent was then rinsed with ultrapure water, dried at ambient temperature, and reused under identical extraction conditions. As illustrated in Figure 14, the recovery values of Cu­(II), Cd­(II), and As­(III) slightly declined over three consecutive reuse cycles, from 95.2 to 88.9%, 94.6 to 87.4%, and 93.0 to 85.6%, respectively. These moderate reductions (approximately 6–8%) are consistent with findings reported in the literature and confirm that the sorbent retains effective extraction capacity for up to three uses. Beyond this point, performance loss due to potential active site degradation or incomplete regeneration may compromise analytical accuracy, necessitating sorbent replacement. Overall, the sorbent demonstrated acceptable reusability, which supports its suitability for cost-effective trace-level environmental monitoring.^117^

**Figure 14:**
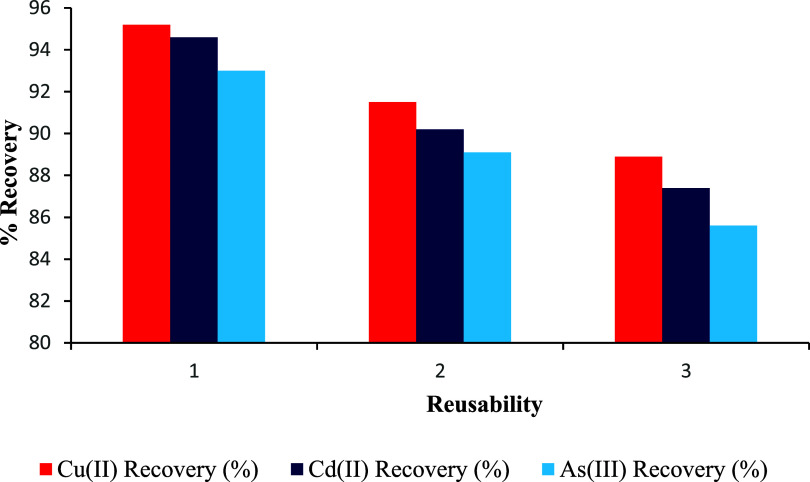
Reusability performance of the TSC-functionalized silica sorbent over three consecutive extraction–desorption cycles for Cu­(II), Cd­(II), and As­(III) ions using the UA-DMSPE method.

### Analytical Performance

The analytical performance of the developed UA-DMSPE-ICP-OES method was rigorously evaluated based on linearity, sensitivity, and precision metrics for Cu­(II), Cd­(II), and As­(III). As summarized in Table 3, excellent linearity was achieved with determination coefficients (*R* ^2^) of 0.9991 for Cu­(II), 0.9984 for Cd­(II), and 0.9987 for As­(III) over the respective concentration ranges of 2–500, 3–500, and 5–500 μg L^–1^. Calibration equations exhibited strong slopes and minimal intercepts, indicating reliable quantification within the selected ranges. The method showed high sensitivity, with limits of detection (LOD) calculated as 0.07 μg L^–1^ for Cu­(II), 0.24 μg L^–1^ for Cd­(II), and 0.30 μg L^–1^ for As­(III), while the corresponding limits of quantification (LOQ) were 0.23, 0.79, and 1.0 μg L^–1^, respectively. LOD and LOQ values were calculated using the standard deviation (Sb) of seven replicate measurements at the lowest point of the linear range, applying the formulas LOD = 3Sb/*m* and LOQ = 10Sb/*m*, respectively, where *m* denotes the slope of the calibration curve. Precision was evaluated by calculating the relative standard deviation (RSD, %) of replicate measurements (*n* = 7) at two concentration levels on the same day (intraday) and across different days (interday).

**Table 3:** Results of Validation of the Proposed Method

parameter	Cu(II)	Cd(II)	As(III)
linear range (μg L^–1^)	2–500	3–500	5–500
calibration equation	*y* = 12836*x* + 1624	*y* = 11542*x* + 2085	*y* = 1391*x* + 1872
*R* ^2^ (*n* = 5)	0.9991	0.9984	0.9987
LOD (μg L^–1^)	0.07	0.24	0.30
LOQ (μg L^–1^)	0.23	0.79	1.0
intraday precision (10 μg L^–1^, %RSD, *n* = 7)	1.35	1.68	1.93
intraday precision (50 μg L^–1^, %RSD, *n* = 7)	1.28	1.52	1.75
interday precision (10 μg L^–1^, %RSD, *n* = 7)	1.62	1.85	2.04
interday precision (50 μg L^–1^, %RSD, *n* = 7)	1.49	1.71	1.98

Precision studies demonstrated consistent and reproducible performance, with intraday relative standard deviations (%RSD) ranging from 1.28 to 1.93% and interday %RSD values between 1.49 and 2.04% at both 10 and 50 μg L^–1^ spiking levels. These results confirm the robustness, accuracy, and applicability of the proposed method for trace-level determination of Cu­(II), Cd­(II), and As­(III) in complex environmental samples.

### Evaluation of Matrix Effects and Method Applicability

The developed UA-DMSPE-ICP-OES method was further validated by evaluating its applicability in complex real matrices including soil, tomato, and water samples. As shown in Table 4, the recovery values for Cu­(II), Cd­(II), and As­(III) ranged from 94.26 to 99% across all matrices, with standard deviations remaining below ±3.13, confirming the method’s high accuracy and reproducibility under realistic sample conditions.

**Table 4:** Recovery and Matrix Effect Evaluation in Water, Soil, and Tomato Samples (*n* = 3)

metal ion	spiked (μg L^–1^)	water measured (μg L^–1^ ± SD)	water recovery (%)	water matrix effect (μg L^–1^ ± SD)	water % diff	soil measured (μg L^–1^ ± SD)	soil recovery (%)	soil matrix effect (μg L^–1^ ± SD)	soil % diff	tomato measured (μg L^–1^ ± SD)	tomato recovery (%)	tomato matrix effect (μg L^–1^ ± SD)	tomato % diff
Cu(II)	20	19.91 ± 2.15	99	21.16 ± 2.15	–2.83%	19.15 ± 2.33	97.32	20.06 ± 2.33	0.13%	18.57 ± 1.7	97.58	17.9 ± 1.7	0.06%
Cu(II)	50	48.14 ± 2.71	96.18	49.36 ± 2.71	–0.21%	49.27 ± 1.75	97.02	47.86 ± 1.75	0.24%	49.39 ± 1.85	99	49.6 ± 1.85	–0.53%
Cd(II)	20	18.6 ± 2.41	95.59	17.4 ± 2.41	1.41%	19.61 ± 2.71	96.72	18.75 ± 2.71	–2.14%	18.59 ± 3.01	96.69	19.25 ± 3.01	3.82%
Cd(II)	50	48.47 ± 2.84	94.26	44.21 ± 2.84	0.97%	47.0 ± 2.48	98.41	47.69 ± 2.48	0.72%	47.26 ± 3.13	97.35	46.47 ± 3.13	0.27%
As(III)	20	18.59 ± 2.57	96.11	20.96 ± 2.57	0.45%	19.18 ± 2.01	96.29	18.52 ± 2.01	2.42%	19.01 ± 1.9	98.47	19.29 ± 1.9	–3.45%
As(III)	50	47.95 ± 2.45	97.14	46.81 ± 2.45	–2.14%	47.71 ± 2.92	96.69	47.1 ± 2.92	–3.0%	48.08 ± 2.53	97.6	46.68 ± 2.53	1.1%

Matrix effect values were calculated by comparing the analyte signals in spiked matrix samples to those in pure standard solutions. The percentage differences (%Diff) remained within the acceptable ±5% range for all three analytes in all matrices, indicating negligible signal suppression or enhancement. Specifically, the tomato matrix yielded recovery values from 96.28 to 98.14% with %Diff values between −2.67 and +1.65%, underscoring the method’s compatibility with complex organic-rich food samples.

No detectable concentrations of Cu­(II), Cd­(II), or As­(III) were found in unspiked real samples, confirming both the selectivity of the method and the absence of background contamination. These results collectively demonstrate the high analytical performance, matrix tolerance, and suitability of the developed method for trace-level determination of toxic metals in environmental and agricultural samples, such as soil and tomato.

### Comparison of the Suggested Method with Other Methods

The analytical performance of the proposed UA-DMSPE-ICP-OES method was systematically compared with those of other microextraction-based techniques previously reported for the determination of trace metals in environmental matrices. As summarized in Table 5, the method demonstrated remarkably low limits of detection (LOD) for Cu­(II), Cd­(II), and As­(III) at 0.07, 0.24, and 0.30 μg L^–1^, respectively. These values are significantly lower than those reported in several earlier studies utilizing similar extraction platforms and in some cases even comparable to more sophisticated techniques such as ICP-MS. Likewise, the LOQ values of 0.23 μg L^–1^ for Cu­(II), 0.79 μg L^–1^ for Cd­(II), and 1.0 μg L^–1^ for As­(III) highlight the method’s suitability for trace-level detection without requiring elaborate sample pretreatment or high-cost instrumentation.

**Table 5:** Comparison of the Developed UA-DMSPE Method with Reported Techniques for Metal Analysis

method	metal	linear range (μg/L)	LOD (μg/L)	LOQ (μg/L)	%RSD	real samples	recovery (%)	refs
UA-DMSPE	Cu(II)	Cu(II): 2–500	Cu(II): 0.07	Cu(II): 0.23	Cu(II): 1.62	water, soil, tomato	94.26–99	this work
	Cd(II)	Cd(II): 3–500	Cd(II): 0.24	Cd(II): 0.79	Cd(II): 1.85			
	As(II)	As(II): 5–500	As(II): 0.30	As(II): 1.0	As(II): 2.04			
DMSPE	Cd(II)		Cd: 0.001		Cd: 3	seawater, lake water, mine water, tap water	95	^108^
	Pb(II)		Pb: 0.03		Pb: 4			
DMSPE	Cd(II)	Cd, Cu, Mn: 1–1000	Cd(II): 0.24	Cd(II): 0.79	Cd(II): 2.71	pork liver kidney	Cd(II): 92.5	^103^
	Pb(II)	Fe: 3–1000	Pb(II): 0.22	Pb(II): 0.73	Pb(II): 3.15		Pb(II): 93.7	
	Zn(II)	Zn: 0.5–1000	Zn(II): 0.035	Zn(II): 0.12	Zn(II): 2.19		Zn(II): 100	
	Fe(II)		Fe(II): 0.84	Fe(II): 2.80	Fe(II): 3.48		Fe(II): 99.7	
	Mn(II)		Mn(II): 0.17	Mn(II): 0.57	Mn(II): 2.90		Mn(II): 100	
SPE	Cu(II)	Cu(II), Ni(II): 50–1000	Cu(II): 0.74		Cu(II): 2.25	water, soil	Cu(II), Ni(II): 90–106	^109^
	Ni(II)		Ni(II): 0.52		Ni(II): 1.72			
SPE	Cr(III)		Cr(III): 0.69	Cr(III): 3.6	Cr(III): 3.7	water	91.4–103.5	^110^
DSPE	Co(II)		Co(II): 0.11	Co(II): 0.36	Co(II): 1.6	water	Co(II): 95.1–103	^111^
	Pb(II)		Pb(II): 0.24	Pb(II): 0.82	Pb(II): 1.2		Pb(II): 93.9–105	
DMSPE	Cr(II)	1–200	Cr(II): 0.11	Cr(II), Co(II), Ni(II), Cu(II) Zn(II), Pb(II): 3.3		water	Cr(II): 101.5	^112^
	Co(II)		Co(II): 0.12				Co(II): 97.6	
	Ni(II)		Ni(II): 0.10				Ni(II): 96.7	
	Cu(II)		Cu(II): 0.07				Cu(II): 98.5	
	Zn(II)		Zn(II): 0.08				Zn(II): 102.9	
	Pb(II)		Pb(II): 0.09				Pb(II): 96.4	
UA-DMSPE	Cd(II)	Cd(II): 0.01–5	Cd(II): 0.0005		Cd(II): 4	water		^113^
	Pb(II)	Pb(II): 0.1–10	Pb(II): 0.01		Pb(II): 5			
US-DMSPE	Mn(II)	Mn(II): 0.03–48.7	Mn(II): 0.007	0.03 (Mn II)	Mn(II): 2.3	water	Mn(II): 102.3	^114^
	Mn(VII)	Mn(VII): 0.04–50.4	Mn(VII): 0.008	0.04 (Mn VII)	Mn(VII): 2.8		Mn(VII): 98.8	
MSPE	Cd(II)	Cd(II): 0.08–1.4	Cd(II): 0.002	Cd(II): 0.008	Cd(II): 4.4–6.1	water, food	Cd(II): 97.2–104	^115^
	Pb(II)	Pb(II): 0.6–30	Pb(II): 0.18	Pb(II): 0.6	Pb(II): 2.9–4.9		Pb(II):98–107	
DMSPE	Cu(II),	1.0–150 (Cu), 0.5– 100 (Cd)	Cu: 0.37, Cd: 0,29	Cu: 1.0, Cd: 0.25	Cu(II): 4.6	water	91.6–98.3	^116^
	Cd(II)				Cd(II): 4.2			

While voltammetric or ICP-MS methods may offer detection in the ng L^–1^ range, these often require advanced technical expertise and rigorous matrix removal and are limited in multielement capability. In contrast, the present method provides a practical and accessible alternative by combining the advantages of ultrasound-assisted dispersive microsolid-phase extraction (UA-DMSPE) with ICP-OES, which allows for simultaneous multielement analysis with excellent reproducibility and matrix tolerance.

Moreover, the proposed method offers a wide linear dynamic range (2–500 μg L^–1^ for Cu­(II), 3–500 μg L^–1^ for Cd­(II), and 5–500 μg L^–1^ for As­(III)) and high recovery rates (94.26–99.0%) across complex matrices such as soil, tomato, and water. Beyond these performance metrics, the distinct advantage of my method stems from the covalent immobilization of the newly synthesized thiosemicarbazone derivative onto silica, which ensures exceptional structural stability, high selectivity toward soft metal ions, and consistent performance across multiple extraction cycles. Unlike many nanoparticle- or polymer-based sorbents reported in the literature, which are prone to leaching, loss of activity, or single-use limitations, the present sorbent retains its integrity and reusability without compromising its recovery. This innovation not only enhances analytical robustness but also underscores the novelty of integrating a purpose-designed ligand into a UA-DMSPE platform, thereby offering a reliable, cost-effective, and environmentally compatible solution for trace metal monitoring in complex matrices. These attributes demonstrate the robustness and versatility of the method, confirming its superiority over many previously published approaches, particularly in terms of sensitivity, precision, operational simplicity, and real-sample applicability.

Therefore, the developed UA-DMSPE-ICP-OES method not only meets but exceeds the performance characteristics required for routine trace metal monitoring in diverse environmental- and food-related matrices, offering a compelling balance between analytical power and practical implementation.

### Molecular Docking

Figures 15,16, and 17 collectively illustrate the binding pockets associated with the lowest docking energy values, emphasizing the molecular interactions between specific protein regions and their corresponding ligands. The accompanying 2D interaction diagrams further elucidate the binding orientations and highlight the key amino acid residues involved, thereby providing a comprehensive visualization of the protein–ligand recognition process.^117–126^ Cavities with the lowest Vina docking scores generally represent favorable binding sites. The predicted binding cavities obtained through the CB-Dock2 platform are comprehensively summarized in Tables 6, 7, and 8. Vina binding energies (kcal/mol), void volumes (Å^3^), and interacting residues are reported for each protein–ligand complex. To facilitate comparative interpretation, a heat map of the docking data was generated in Microsoft Excel. As seen in Figure 18, the strongest binding affinity, indicated by the most negative docking energy, was observed in the interaction between the compound TSC and the 3KFD protein target. In general, docking energies below −5.0 kcal/mol are considered indicative of strong ligand–protein binding affinity.^127^

**Figure 15:**
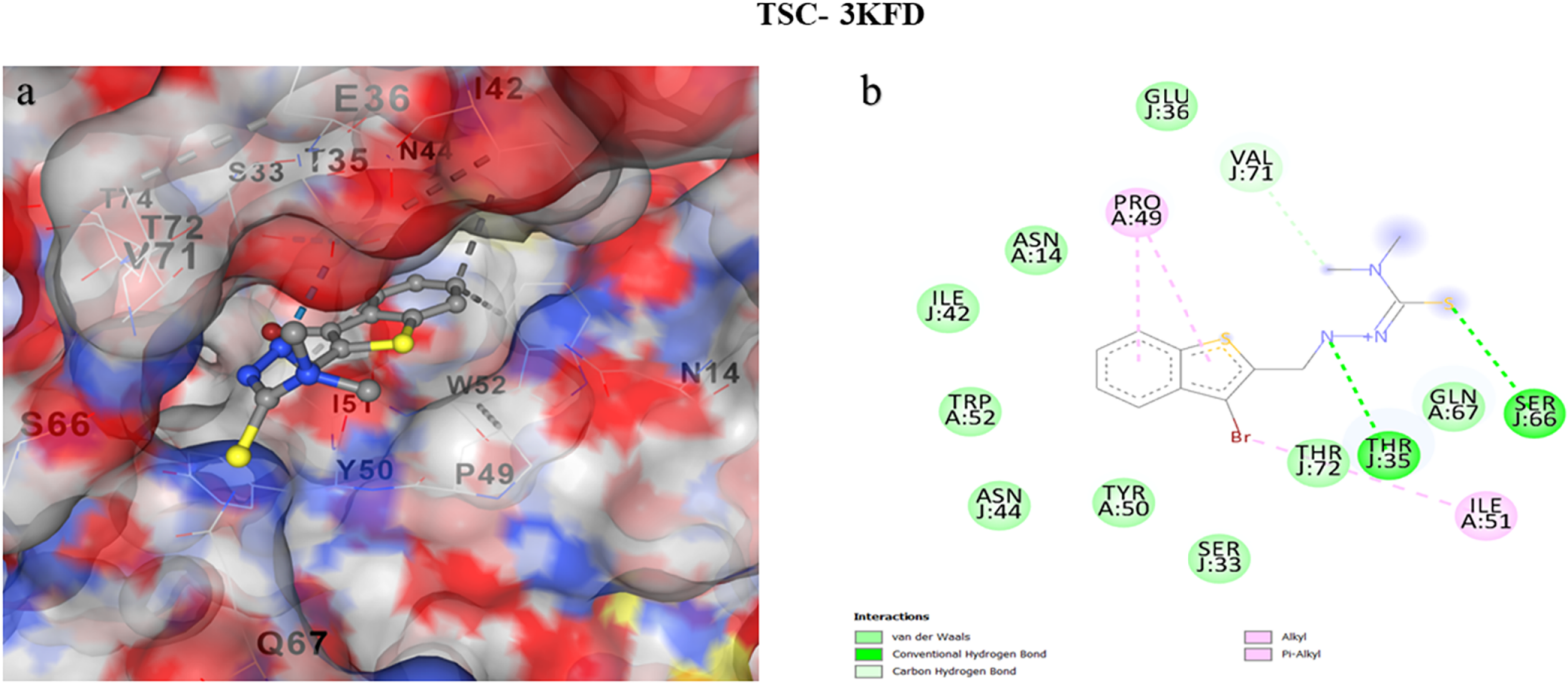
(a) Molecular docking images of cavities with the highest binding affinities between TSC and 3KFD, (b) 2D interaction images of cavities with the highest binding affinities between TSC and 3KFD.

**Figure 16:**
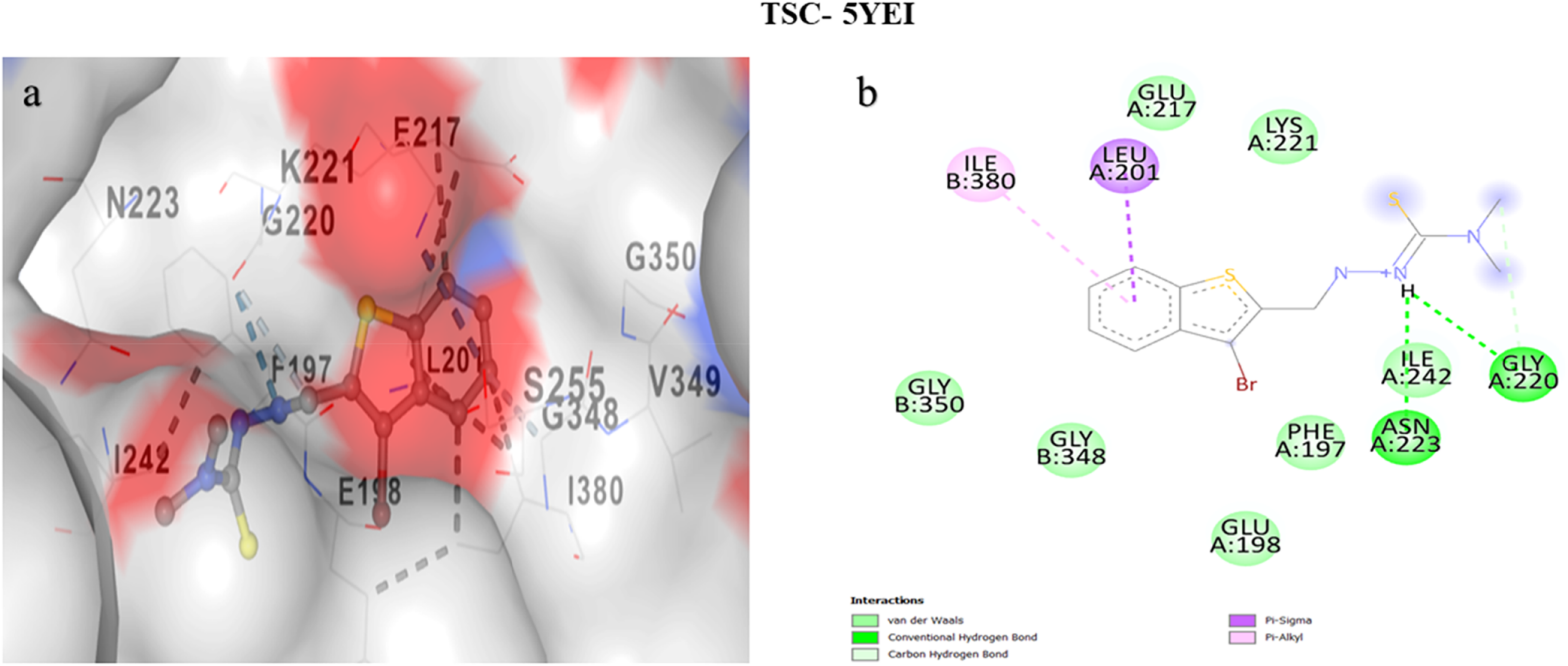
(a) Molecular docking images of cavities with the highest binding affinities between TSC and 5YEI, (b) 2D interaction images of cavities with the highest binding affinities between TSC and 5YEI.

**Figure 17:**
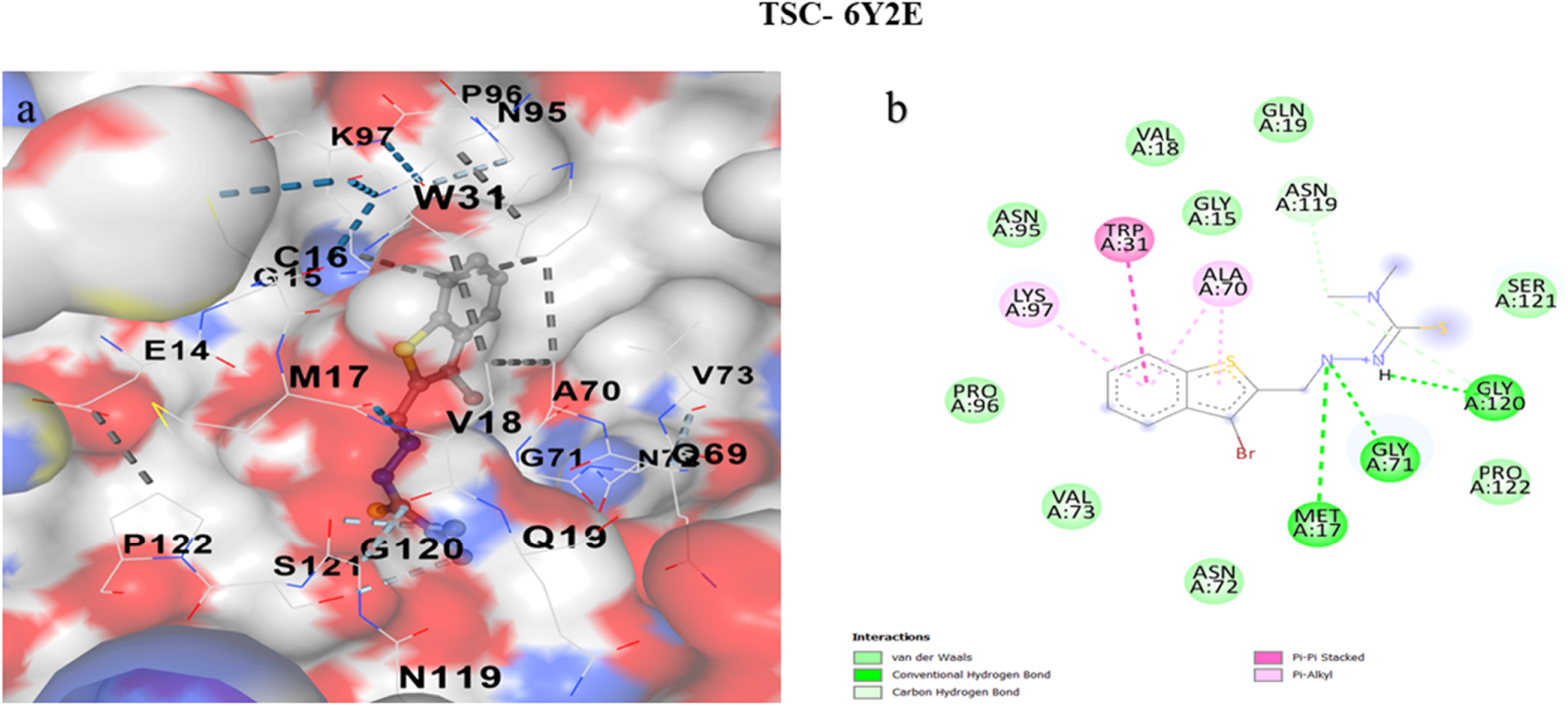
(a) Molecular docking images of cavities with the highest binding affinities between TSC and 6Y2E, (b) 2D interaction images of cavities with the highest binding affinities between TSC and 6Y2E.

**Figure 18:**
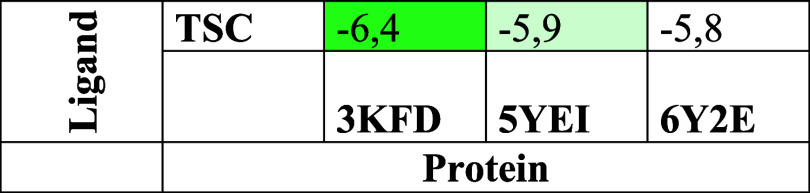
Molecular docking heat map of proteins and ligands (binding affinities are displayed by color: The darker color means a lower binding energy, which indicates better binding).

**Table 6:** Molecular Docking Results of TSC with 3KFD

receptor (protein)	CurPocket ID	Vina score (kcal/mol)	cavity volume (Å^3^)	center (*x*, *y*, *z*)	docking size (*x*, *y*, *z*)	contact residues
3KFD	C4	–6.4	1487	–15, −72, −34	21, 21, 21	**Chain A:** TYR6, SER10, THR11, GLU12, LYS13, ASN14, PRO49, TYR50, ILE51, TRP52, ASN66, GLN67, PRO70, GLY71
						**Chain J:** PHE13, HIS15, PHE22, SER33, THR35, GLU36, THR37, VAL41, ILE42, HIS43, ASN44, SER65, SER66, VAL71, THR72, THR74

**Table 7:** Molecular Docking Results of TSC with 5YEI

receptor (protein)	CurPocket ID	Vina score (kcal/mol)	cavity volume (Å^3^)	center (*x*, *y*, *z*)	docking size (*x*, *y*, *z*)	contact residues
5YEI	C5	–5.9	1627	37, −63, −12	27, 21, 35	**Chain B:** SER255, ALA258, PHE259, ASN260, ASP262, LYS343, SER345, VAL347, GLY348, VAL349, GLY350, ILE380, LYS381, SER383
						**Chain F:** PHE259, ARG261, GLU392, VAL395, ARG396
						**Chain A:** THR196, PHE197, GLU198, LEU201, VAL216, GLU217, GLY220, LYS221, ASN223, ILE242, ASP243, VAL349, ARG352, SER353, SER378, GLU379, ILE380

**Table 8:** Molecular Docking Results of TSC with 6Y2E

receptor (protein)	CurPocket ID	Vina score (kcal/mol)	cavity volume (Å^3^)	center (*x*, *y*, *z*)	docking size (*x*, *y*, *z*)	contact residues
6Y2E	C3	–5.8	250	–7, −47, 10	21, 21, 21	**Chain A:** GLU14, GLY15, CYS16, MET17, VAL18, GLN19, TRP31, GLN69, ALA70, GLY71, ASN72, VAL73, LEU75, THR93, ASN95, PRO96, LYS97, TYR118, ASN119, GLY120, SER121, PRO122

The selection of protein targets for molecular docking was guided by their biological relevance to diverse therapeutic contexts where thiosemicarbazone (TSC) scaffolds have shown promise. The 3KFD structure represents a neuroreceptor-related model protein, offering insights into TSC interactions with neuronal targets that can be potentially linked to neurodegenerative pathways. Aspartate kinase (5YEI) was chosen as a representative metabolic enzyme, where inhibition may perturb microbial or cancer cell growth through the interference with amino acid biosynthesis. Finally, the SARS-CoV-2 main protease (6Y2E) serves as a clinically significant viral enzyme, enabling exploration of the antiviral potential for TSC derivatives. Together, these proteins provide a cross section of neurological, metabolic, and viral targets, thereby highlighting the structural adaptability and pharmacological versatility of TSC ligands.^128^

Blind docking located low-energy binding cavities for TSC in all three proteins, with Vina scores spanning −6.4 to −5.8 kcal mol^–1^ (3KFD C4:6.4; 5YEI C5: −5.9; 6Y2E C3: −5.8). These values fall within the range generally interpreted as moderate, specific binding in structure-based screening studies of thiosemicarbazone scaffolds.^128–130^ In the context of TSCs, such scores are consistent with stabilizing combinations of hydrogen bonding to heteroatom donors/acceptors (C=S/C=N region) and π-type contacts to aromatic residues, as reported for closely related ligands.^128,131^

3KFD (pocket C4, 1487 Å^3^): The pose engages a mixed polar–aromatic environment formed by residues from two chains (A, J). The contact set includes polar/charged residues (SER10, THR11, GLU12, LYS13, ASN14, ASN66, GLN67, SER33, THR35–37, GLU36) that can sustain a hydrogen-bond/salt-bridge network to the TSC azomethine N and thione S, alongside multiple aromatics (TYR6/50, TRP52, PHE13/22) providing π–π or edge-to-face stabilization to the ligand’s conjugated system. Hydrophobic side chains (ILE51–52, VAL41, ILE42, and VAL71) further buttress the complex through dispersion contacts. The combination of polar anchoring and aromatic stacking rationalizes the most favorable score in the series (−6.4 kcal mol^–1^) and matches interaction motifs commonly observed for TSCs in enzyme pockets.^128,129,132,133^

5YEI (Aspartate kinase; pocket C5, 1627 Å^3^): Despite the larger cavity, the predicted affinity (−5.9 kcal mol^–1^) remains competitive, supported by a dense array of H-bond donors/acceptors (SER255, ASN260, ASP262, SER345, SER353, SER378, GLU198/217/379/392) that can pair with the ligand’s imine nitrogen and thione sulfur. Basic residues (LYS343/381/221, ARG352/396) are positioned to form salt bridges or cation−π contacts with the ligand’s π-system, while multiple aromatics (PHE197/259) can engage π–π stacking. This polar–aromatic complementarity is characteristic for TSC binding and aligns with prior docking studies that attribute TSC stabilization to concurrent H-bonding and π interactions in kinases and oxidoreductases.^129,131,134^

6Y2E (SARS-CoV-2 Mpro; pocket C3, 250 Å^3^): The smallest cavity nonetheless supports a similar score (−5.8 kcal mol^–1^), suggesting a snug, shape-complementary fit. The pose is framed by GLU14/GLN19/ASN72/ASN95/ASN119/GLY–SER motifs that can hydrogen bond to the C=N/C=S region together with TRP31 and TYR118 that provide π-stacking or CH−π stabilization. Nearby LYS97 and hydrophobic residues (VAL18/73, LEU75, ALA70, and PRO96/122) contribute electrostatic and dispersion contacts. The balance of polar anchors and aromatic contacts mirrors binding patterns reported for heteroaromatic TSCs docked to proteases and supports a plausible, albeit moderate, interaction at an allosteric or peripheral site.^127,130,133,135^

Across the panel, the best score at 3KFD (−6.4 kcal mol^–1^) coincides with a cavity rich in both H-bond partners and aromatics, while 5YEI (−5.9 kcal mol^–1^) benefits from a large, polar pocket with accessible basic and aromatic residues; 6Y2E (−5.8 kcal mol^–1^) appears to be driven by steric complementarity and mixed polar/π contacts in a compact site. These patterns are fully consistent with the donor–acceptor profile of thiosemicarbazones—where the azomethine N and thione S act as primary H-bonding/coordination loci and the conjugated backbone participates in π-stacking—and with docking precedents for TSC frameworks showing similar energy windows and contact topologies.^128,129,132–134^

Previous studies on thiosemicarbazone derivatives have reported docking energies and interaction profiles characterized by multiple hydrogen bonds to heteroatoms, π–π stacking interactions with aromatic residues such as Phe, Tyr, and Trp, and occasional cation−π or salt-bridge interactions with Lys and Arg. These interaction motifs closely align with the present findings, reinforcing the chemical plausibility of the predicted binding poses and supporting the moderate affinity range observed in this work.^127–129,132,133,135^

### DFT Analysis

Density functional theory (DFT) calculations were carried out to gain insight into the electronic structure and reactivity profile of the synthesized thiosemicarbazone (TSC) derivative (Figure 19). As shown in Figure 20, the frontier molecular orbital distribution and the energy gap between the highest occupied molecular orbital (HOMO, −7.49 eV) and the lowest unoccupied molecular orbital (LUMO, −5.39 eV) were computed, revealing an energy gap (Δ*E*) of 2.10 eV. This moderate band gap suggests a balanced interplay between molecular stability and reactivity, with potential for chelation and electron-transfer interactions with heavy metal ions such as Cu­(II), Cd­(II), and As­(III).^133,136^

**Figure 19:**
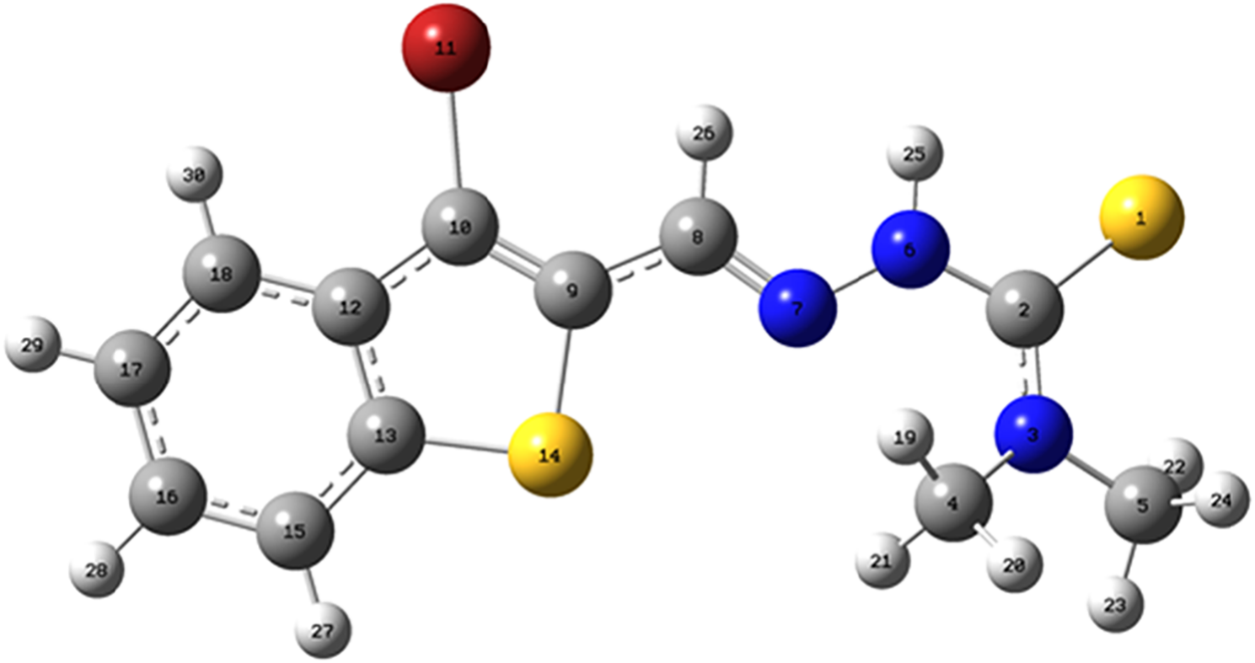
Optimized geometries of the TSC.

**Figure 20:**
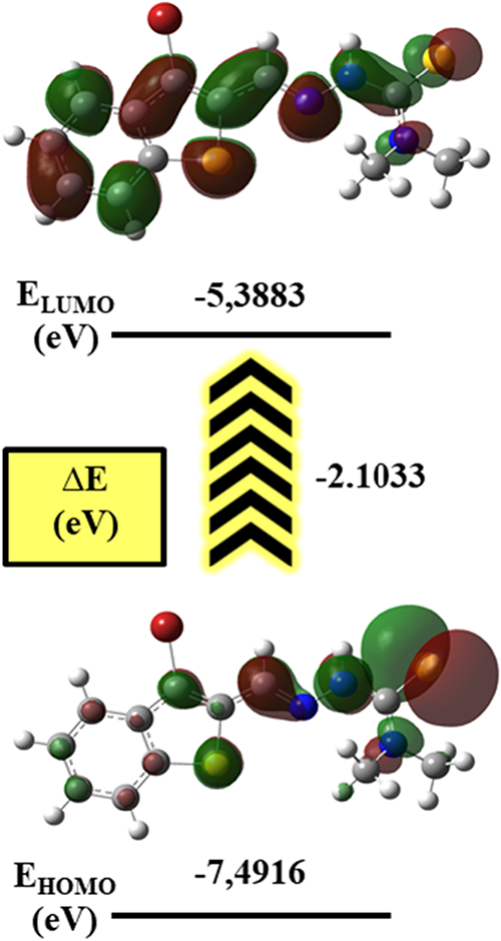
Frontier molecule orbitals and energy gaps of the TSC.

The spatial distribution of the frontier molecular orbitals (Figure 20) reveals key insights into the electronic properties and reactivity of the synthesized thiosemicarbazone (TSO) ligand. The highest occupied molecular orbital (HOMO) is predominantly localized over the thiosemicarbazone core, especially around the azomethine nitrogen (N7) and thione sulfur (S14) atoms. In contrast, the lowest unoccupied molecular orbital (LUMO) is delocalized toward the aromatic ring system and the carbonyl oxygen atom (O11), suggesting that the electron-accepting capability is concentrated in these π-rich and electronegative regions.

This electron distribution pattern implies that the electron-donating atoms particularly N7 and S14 are well-positioned for interaction with soft metal centers, thereby enhancing the ligand’s chelating efficiency in complexation-based extraction processes.

Further insights are provided by the molecular electrostatic potential (MEP) surface (Figure 21), which illustrates the regions of electron density across the molecule. The most negative electrostatic potential (depicted in red) is concentrated around the carbonyl oxygen (O11) and thione sulfur (S14) atoms, confirming their strong nucleophilic character and their critical role in metal coordination.^137^ Conversely, the most electropositive regions (shown in blue) are associated with the N–H hydrogen atoms (e.g., H25 and H26) on the hydrazine portion, consistent with their potential involvement in hydrogen bonding or proton transfer interactions.

**Figure 21:**
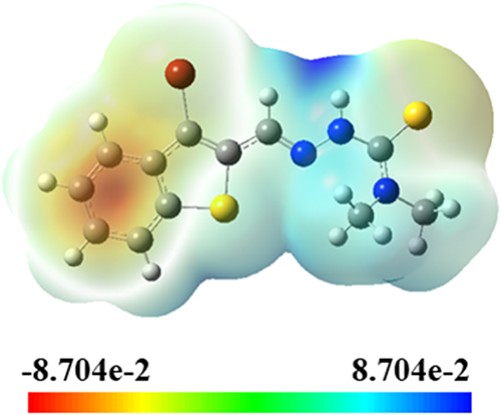
Molecular electrostatic potential maps of TSC.

The calculated global reactivity descriptors are summarized in Table 9. The ionization potential (IP) and electron affinity (EA) were derived from the HOMO and LUMO energies, yielding values of 7.49 and 5.39 eV, respectively. Based on these values, the electronegativity (χ = 6.44 eV), global hardness (η = 1.05 eV), and softness (*S* = 0.476 eV^–1^) were calculated. These parameters suggest that the compound possesses a moderate electrophilic character with sufficient chemical softness to undergo nucleophilic or electrophilic interactions. The global electrophilicity index (ω = 19.76 eV), which quantifies the stabilization energy gained upon accepting electrons, further supports the compound’s potential as a reactive ligand in coordination chemistry.^138–140^

**Table 9:** Chemical Parameters of TSC

chemical parameters	TSC derivative
*E*_HOMO_	–74,916
*E*_LUMO_	–53,883
Δ*E*_energy gap_	–21,033
ionization potential (IP)	74,916
electron affinity (EA)	53,883
electronegativity (χ)	64,400
hardness (η)	10,517
softness (σ)	352,0142
chemical potential (μ)	–64,400
global electrophilicity (ω)	19,7173
*E*_0_	140.95 kcal/mol
Δ*H* (298 K)	152.75 kcal/mol
Δ*G* (298 K)	110.41 kcal/mol

Thermodynamic parameters derived from the vibrational frequency analysis further substantiate the stability and feasibility of the synthesized thiosemicarbazone derivative. The absence of imaginary frequencies confirms that the optimized molecular structure corresponds to a true energy minimum. The zero-point-corrected electronic energy (*E*_0_) was calculated as 140.95 kcal/mol. Additionally, the enthalpy change (Δ*H*) and Gibbs free energy (Δ*G*) at 298 K were determined to be 152.75 and 110.41 kcal/mol, respectively. These moderately exothermic values indicate that the formation of the compound is thermodynamically favorable. The significantly negative Gibbs free energy further supports the spontaneity of the synthesis process under ambient conditions. Overall, the computed thermodynamic data validate both the energetic stability and the synthetic accessibility of the designed thiosemicarbazone ligand.^141^

Importantly, these theoretical findings provide a direct explanation for the experimentally observed high selectivity and strong binding of the ligand to Cu­(II), Cd­(II), and As­(III). The localization of HOMO on the azomethine nitrogen and thione sulfur, together with the intense negative electrostatic potential around these donor atoms, confirms their role as preferential coordination sites for soft metal ions. The relatively narrow HOMO–LUMO gap further indicates a favorable electron-transfer process during complexation, while the high electrophilicity index supports the ligand’s ability to stabilize metal–ligand interactions. Thus, the DFT calculations not only validate the structural and energetic suitability of the ligand but also rationalize its superior extraction performance, demonstrated in UA-DMSPE experiments.

Taken together, these theoretical insights affirm that the synthesized TSC derivative exhibits suitable electronic, electrostatic, and thermodynamic properties for effective coordination with heavy metal ions, thus supporting its application in microextraction procedures targeting trace metal analysis in complex matrices such as soil, water, and tomato samples.

## Conclusions

In this study, a novel thiosemicarbazone derivative was successfully synthesized and characterized using spectroscopic (FTIR, NMR) techniques. Its coordination behavior toward Cu­(II), Cd­(II), and As­(III) ions was systematically investigated using the UA-DMSPE method, demonstrating high extraction efficiency and selectivity under optimized conditions. The method was effectively applied to real samples, including water, soil, and tomato matrices, highlighting its potential for environmental and food safety monitoring.

Computational studies provided theoretical insights into the stability and electronic features of the ligand with DFT calculations supporting and rationalizing the experimentally observed donor–acceptor interactions. Molecular docking analyses revealed moderate but specific binding affinities of the ligand toward biologically relevant proteins, suggesting potential pharmacological relevance in addition to its analytical applications.

Overall, the integration of synthesis, analytical validation, and computational modeling provides comprehensive insight into the multifunctional properties of this novel thiosemicarbazone derivative. The findings not only establish a reliable platform for trace metal monitoring in complex matrices but also open avenues for future investigations of thiosemicarbazones as dual-purpose ligands with both environmental and biomedical significance.
